# Fast Coalescent-Based Computation of Local Branch Support from Quartet Frequencies

**DOI:** 10.1093/molbev/msw079

**Published:** 2016-04-15

**Authors:** Erfan Sayyari, Siavash Mirarab

**Affiliations:** ^1^Department of Electrical and Computer Engineering, University of California at San Diego

**Keywords:** Incomplete lineage sorting, multi-species coalescent, quartet-based methods, ASTRAL, posterior probability, local support, branch length estimation.

## Abstract

Species tree reconstruction is complicated by effects of incomplete lineage sorting, commonly modeled by the multi-species coalescent model (MSC). While there has been substantial progress in developing methods that estimate a species tree given a collection of gene trees, less attention has been paid to fast and accurate methods of quantifying support. In this article, we propose a fast algorithm to compute quartet-based support for each branch of a given species tree with regard to a given set of gene trees. We then show how the quartet support can be used in the context of the MSC to compute (1) the local posterior probability (PP) that the branch is in the species tree and (2) the length of the branch in coalescent units. We evaluate the precision and recall of the local PP on a wide set of simulated and biological datasets, and show that it has very high precision and improved recall compared with multi-locus bootstrapping. The estimated branch lengths are highly accurate when gene tree estimation error is low, but are underestimated when gene tree estimation error increases. Computation of both the branch length and local PP is implemented as new features in ASTRAL.

## Introduction

The multi-species coalescent model (MSC) of Rannala and Yang (2003) has emerged as the standard method used for reconstructing species trees in the presence of gene tree discordance due to incomplete lineage sorting (ILS) (Maddison 1997; Degnan and Rosenberg 2006). Many methods have been developed to estimate species trees under the MSC (e.g., Heled and Drummond 2010; Bryant et al. 2012; Chifman and Kubatko 2014; Mirarab, Bayzid, Boussau, et al. 2014). The most scalable family of MSC-based methods are based on a two-step process where gene trees are first estimated independently for each gene and are then combined to build the species tree using a *summary method*. Many of the summary methods are statistically consistent and thus converge in probability to the true species tree as the number of input error-free gene trees increases; examples of consistent methods include ASTRAL (Mirarab, Reaz, Bayzid, et al. 2014; Mirarab and Warnow 2015), BUCKy-population (Larget et al. 2010), GLASS (Mossel and Roch 2010), MP-EST (Liu et al. 2010), NJst and ASTRID (Liu and Yu 2011; Vachaspati and Warnow 2015), and STAR (Liu et al. 2009). While some methods (e.g., MP-EST) can estimate branch lengths in coalescent units, others only infer the topology. The traditional concatenation approach (where all genes are put together in a supermatrix) can produce high support for incorrect branches (Kubatko and Degnan 2007; Roch and Steel 2014), and the main goal of statistically consistent summary methods is to address this shortcoming. However, despite the progress in developing methods for species tree reconstruction, little attention has been paid to methods of calculating support.

Bayesian methods (e.g., Liu 2008; Heled and Drummond 2010) readily provide support but remain computationally challenging. Calculating support through bootstrapping (Felsenstein 1985), while still computationally expensive, is not prohibitively slow and is easily parallelizable. Seo et al. (2005) proposed a multi-locus bootstrapping (MLBS) procedure that produces bootstrap replicates by first resampling genes and then sites within those sampled genes. Seo (2008) later studied the accuracy of the MLBS approach in the context of distance-based tree reconstruction for 4-taxon trees and explored other strategies where only genes or only sites were resampled. These earlier works did not consider ILS as the cause of discordance; nor did they use summary methods. Nevertheless, the community has adopted MLBS as a standard way of estimating support using ILS-based summary methods; most biological studies using summary methods rely on site-only or site/gene MLBS (e.g., Song et al. 2012; Jarvis et al. 2014; Wickett et al. 2014; Prum et al. 2015).

Recently, Mirarab, Bayzid, Warnow (2014) studied the reliability of MLBS support values as a measure of accuracy in simulation studies, and documented both under-estimation and over-estimation of support (for low- and high-support branches, respectively) using MP-EST (Liu et al. 2010) and supertree methods such as MRP (Ragan 1992) and MRL (Nguyen et al. 2012). Mirarab, Bayzid, Warnow (2014) also observed better species tree accuracy when a summary method was run directly on ML gene trees, compared with running the summary method on bootstrapped gene trees first and then taking a consensus. This observation led to the conjecture that MLBS might give biased estimates of the support. Furthermore, Bayzid et al. (2015) found in simulation studies that false positive branches can sometimes have high MLBS support and that many true branches tend to have low support. Obtaining low support for true branches should not be a cause of concern if the lack of support is caused by insufficient data; however, when low support is caused by underestimation, we need better methods of quantifying support.

In this article, we show that using properties of the MSC on four taxa (quartets), we can derive support values that are more precise and more powerful than MLBS, and are much faster to compute. Under the MSC, quartet trees do not have anomaly zones (Allman et al. 2011; Degnan 2013), meaning that the most probable gene tree is identical to the species tree for any quartet. Exploiting this property, some summary methods break up gene trees into quartet trees. For example, ASTRAL (Mirarab and Warnow 2015), a summary method used in many recent studies (e.g., Wickett et al. 2014; Andrade et al. 2015; Giarla and Esselstyn 2015; Grover et al. 2015; Hosner et al. 2015; Laumer et al. 2015; Prum et al. 2015; Streicher et al. 2015), finds the species tree that shares the maximum number of induced quartet trees with gene trees.

**Fig. 1:**
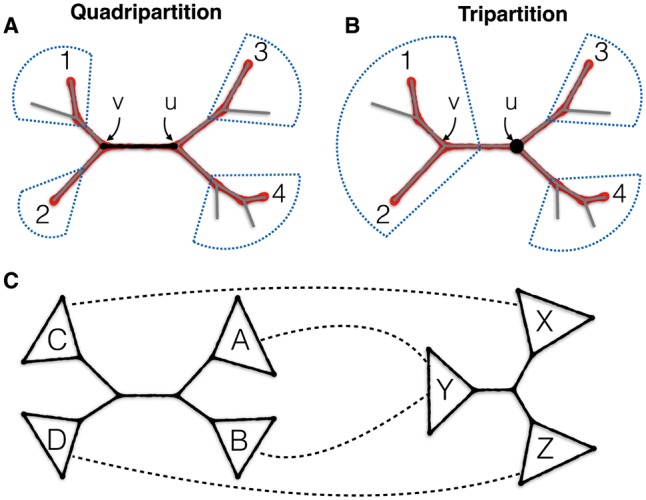
Quadripartitions and tripartitions. (*A*) An internal branch (black, in the middle) divides the set of leaves into a quadripartition. A quartet of leaves {1,2,3,4} induces a quartet tree (in red) with two internal nodes that map to two nodes in the larger tree (here, *u* and *v*). (*B*) An internal node, here *u*, divides leaves into a tripartition; a selection of two leaves from one side and one from each remaining side gives a quartet mapped to that tripartition. Each quartet tree also maps to a second tripartition (*v*). (*C*) An example mapping between a quadripartition (e.g., from the species tree) and a tripartition (e.g., from a gene tree); 12 such mappings exist. Note that by finding all quartets of the form (a,b,c,d);a∈A∩Y,b∈B∩Y,c∈C∩X,d∈D∩Z, we can find all quartets around the quadripartition that are mapped to the tripartition with this mapping.

We introduce a new method for computing support for species tree branches with regard to a set of unrooted gene trees by calculating Bayesian posterior probabilities. Our support values, which we call *local posterior probabilities*, are computed based on gene tree quartet frequencies. For each internal branch of the given species tree, we *assume* that the four *sides* of the branch (fig. 1) are correct, and therefore three topologies are possible around that branch. We introduce a fast algorithm to compute a *quartet support* for each of those three alternatives in Θ(nl) time (where l is the number of species and n is the number of genes). We then use the quartet support for each alternative topology to derive the posterior probability (PP) that it is the correct species topology. Besides producing measures of support, quartet frequencies can be used to derive estimates of internal branch lengths in coalescent units.

Our calculation of posterior probabilities is analogous to characterizing a biased die. If we toss a three-faceted biased die *n* times, our belief in whether the die is biased toward a certain side should depend on the number of tosses and also on the bias of the die (less bias requires more tosses). Similarly, a short branch in the species tree will result in high discordance, and will need many genes to resolve it with high confidence. On the other hand, considering only the MSC and ignoring issues such as long branch attraction, long branches can be easily reconstructed confidently even with few genes.

We show using simulated and empirical datasets that the local PP estimated by our approach is a reliable measure of accuracy. We show that very few highly supported branches are incorrect. Moreover, with a sufficient number of genes, most correct branches have high support. Importantly, we test our methods under conditions where assumptions of our model are violated and show that it remains reliable. Our method is available as part of ASTRAL (https://github.com/smirarab/ASTRAL/), which now estimates species tree topologies, branch lengths, and local posterior probabilities.

## New Approaches

### Definitions

Throughout this article, we only consider unrooted trees. Let L be the set of l leaves (i.e., taxa). Each branch of a tree T creates a bipartition on L, and we say that each of the two partitions is a *cluster* in T. Each *internal* branch divides L into four clusters, creating a quadripartition (fig. 1). Similarly, an internal node divides L into three clusters, creating a tripartition (fig. 1). Any quartet q of taxa induces a quartet tree t on T. The two internal nodes of t correspond to two internal nodes in T. When those two internal nodes are the two sides of a single branch in *T*, we say that the quartet q is *around* that branch. The set of quartets around a given quadripartition can be built by enumerating all selections of one leaf from each of its four clusters.

### Problem Statement

We are given a set of n gene trees evolved on an unknown true binary species tree according to the MSC model. Our aim is to *score* a given internal branch represented as a quadripartition *Q* to estimate:

1. the probability that Q is in the true species tree, assuming clusters of Q are each correct,
2. the length of Q in coalescent units, assuming Q is a correct branch in the species tree.

### Assumptions

We assume that evolution is tree-like and true gene trees differ from the species tree only due to ILS, as modeled by the MSC. We also assume we are given an unbiased sample of true gene trees. On real data, we need to instead estimate gene trees from sequence data, and further, it is not always clear that our sample is unbiased, nor that gene trees are generated by the MSC.

Importantly, we further assume that all four clusters around the branch we are scoring are correct. This assumption, which we refer to as the *locality assumption*, makes our computations tractable for large datasets. Similar assumptions have been made in past for fast calculation of local support in the context of maximum likelihood (ML) tree reconstruction from sequence data; for example, aLRT in PhyML (Guindon et al. 2009) and SH-like support in FastTree-II (Price et al. 2010). Note that to test our method, we use data that violate the locality assumption and we also use estimated gene trees in addition to true gene trees generated by the MSC.

### Calculation of Local Posterior Probability

#### Quartet Trees

Our approach is based on analyzing quartets defined around the branch *Q*. For any quartet of leaves around *Q*, we have three possible topologies, which we will call $t_{1},\;t_{2}$, and $t_{3}$. In the MSC model, the quartet topology found in the true species tree has the highest probability of appearing in gene trees (Allman et al. 2011), and the two alternative topologies have identical probabilities. Furthermore, if the total branch length (in coalescent units) between the two internal nodes of the quartet is *d*, the probability of the dominant quartet topology in gene trees is $\theta =1-\frac{2}{3}e^{-d}>\frac{1}{3}$, and the probabilities of both alternative topologies are $\frac{1-\theta }{2}=\frac{1}{3}e^{-d}<\frac{1}{3}$.

We refer to the number of times $t_{1},t_{2},$ and $t_{3}$ are induced in gene trees as *quartet frequencies*, shown as $\bar{n}=(n_{1},n_{2},n_{3})$; note $\sum_{1}^{3}n_{j}=n$. In the MSC model, conditioned on the species tree, gene trees are independent; Thus, $\bar{n}$ can be modeled as a multinomial random variable $\bar{N}$, with parameters *θ*, $\frac{1-\theta }{2},\;\frac{1-\theta }{2}$, where *θ* corresponds to the species tree topology, and $\frac{1-\theta }{2}$ to the alternative topologies. A similar model is used in the maximum pseudo-likelihood approach of Liu et al. (2010) for triplets.

#### Multiple Quartets

There are $m=\prod_{1}^{4}m_{i}$ quartets around branch *Q*, where *m_i_* is the size of a cluster of the quadripartition of *Q*. Note that we can rearrange clusters of *Q* to obtain two alternative quadripartitions, which we call *Q*_2_ and *Q*_3_. Let ${\bar{n}}_{i}=(n_{1i},n_{2i},n_{3i}),\;1\leq i\leq m$ be quartet frequencies for all *m* quartets around branch *Q* such that $n_{1i},\;n_{2i}$, and $n_{3i}$ correspond to the topologies $Q,\;Q_{2}$, and *Q*_3_, respectively.

Each ${\bar{n}}_{i}$ can be modeled as a multinomial random variables ${\bar{N}}_{i}$, and ${\bar{N}}_{i}$s are identically distributed. To use all ${\bar{n}}_{i}$ values, one approach is to assume they are also all independent, and model $\left({\sum n}_{1i},{\sum n}_{2i},{\sum n}_{3i}\right)$ as observations from a multinomial with m×n trials. The independence assumption would clearly be incorrect; topologies of different quartets around a branch heavily depend on each other (supplementary fig. S15, Supplementary Material online, shows an example). Quartets around a branch are dependent even when the locality assumptions hold. A big problem with the independence assumption is that it inflates confidence because the number of observations (i.e., die tosses) becomes m×n instead of n, thereby greatly increasing posterior probabilities (note m≥l−3). Moreover, the dependence of various quartets on each other is intricate and hard to model.

To avoid inflating posterior values by assuming independence, we take the opposite conservative approach. We assume that a hidden random variable $\bar{Z}$ gives a single vector of “true” quartet frequencies around *Q* and treat each ${\bar{N}}_{i}$ as a noisy estimate of $\bar{Z}$. Thus, $\bar{Z}$ follows a multinomial distribution with n tries (irrespective of *m*) and ${\bar{N}}_{i}=\bar{Z}+\bar{Y}$ for 1≤i≤m where $\bar{Y}$ is a noise term with zero expectation. In the die analogy, we assume the die is tossed n times, and for each toss, we read the outcome *m* times, each time with some noise. Ideally, we should have a noise model and compute the posterior with respect to the given ${\bar{N}}_{i}$ values by marginalizing over $\bar{Z}$. However, a good noise model is not available and the resulting problem becomes hard to solve. Instead, we treat the expected value of $\bar{Z}$ as an observed value, and empirically estimate it by averaging:

(1) $$z_{j}=\frac{\sum_{1}^{m}n_{ji}}{m}\;\;\;\text{for}\;j\in \{1,2,3\}$$

At the end of this section, we will introduce an efficient Θ(nl) algorithm to compute $\bar{z}$.

Lemma 1 Let $\left(\theta_{1},\theta_{2},\theta_{3}\right)$ denote parameters of the true multinomial distribution generating $\bar{Z}$. Note $\sum_{1}^{3}\theta_{i}=1$ and the two lower θ*_i_*s are identical, and recall $z_{1}$ corresponds to the topology of Q.

(2) $$P\left(\theta_{1}>\frac{1}{3}|\bar{Z}=\bar{z}\right)=\frac{\int_{\frac{1}{3}}^{1}P(\bar{Z}=\bar{z}|\theta_{1}=t)f_{\theta_{1}}(t)\text{d}t}{P(\bar{Z}=\bar{z})},$$

where $f_{\theta }$ is the prior PDF. The likelihood term is

(3) $$P(\bar{Z}=\bar{z}|\theta_{1}=t)=\Gamma t^{z_{1}}{\left(\frac{1-t}{2}\right)}^{n-z_{1}},$$

where $\Gamma =\frac{\Gamma (n+1)}{\prod_{1}^{3}\Gamma (z_{j}+1)}$, and marginal probability is

(4) P(Z¯=z¯)=∑j=13∫131P(Z¯=z¯|θj=t)fθj(t)dt=Γ∑j=13∫131tzj(1−t2)n−zjfθj(t)dt.

The proof is given in Supplementary Material online. *Q* is in the species tree iff $\theta_{1}>\frac{1}{3}$; thus, with Lemma 1 and a prior we can compute the PP.

#### Prior

In absence of extra reliable information about the species tree topology, which is the most common scenario, the use of an uninformative prior is justified. An uninformative prior would require that the three topologies are equally likely $\left(\text{i.e., }P\left(\theta_{1}>\frac{1}{3}\right)=P\left(\theta_{2}>\frac{1}{3}\right)=P\left(\theta_{3}>\frac{1}{3}\right)=\frac{1}{3}\right)$. Based on Theorem 3.3 from Stadler and Steel (2012), we can prove (see Supplementary Material online):

Lemma 2 If the species tree is generated using the Yule process with rate λ, branch lengths are exponentially distributed, and for $t\geq \frac{1}{3}$:

(5) $$f_{\theta_{j}}(t)=\lambda {\left(3\frac{1-t}{2}\right)}^{2\lambda -1}.$$

We use (5) throughout the paper as the prior ($\lambda =\frac{1}{2}$ gives a flat prior). Note that we need that branch lengths in *coalescent units* follow properties of the Yule process; this can be achieved if lengths measured by the number of generations follow the Yule process and *N_e_* is constant for all branches.

#### Local PP

We now conclude

Theorem 1 Given (1) a set of n gene trees generated by the MSC on a model species tree generated by the Yule process with rate λ and (2) an internal branch represented by a quadripartition *Q* where the four clusters around *Q* are each present in the species tree, let $\bar{z}=(z_{1},z_{2},z_{3})$ be the average quartet frequencies around *Q* (where $z_{1}$ corresponds to the topology of *Q*); the local PP that the species tree has the topology given by *Q* is:

(6) $$P(Q|\bar{Z}=\bar{z})=\frac{h(z_{1})}{h(z_{1})+2^{z_{2}-z_{1}}h(z_{2})+2^{z_{3}-z_{1}}h(z_{3})}$$

for $h(x)=B(x+1,n-x+2\lambda )(1-I_{\frac{1}{3}}(x+1,n-x+2\lambda ))$. Here, B(α,β) is the beta function, and *I_x_* is the regularized incomplete beta function.

*Proof (sketch)*. With locality assumption, $\bar{Z}$ follows a multinomial distribution with parameters $\left(\theta_{1},\theta_{2},\theta_{3}\right)$. Lack of anomaly zones for unrooted quartets, shown by Allman et al. (2011) means that *Q* is in the species tree iff $\theta_{1}>\frac{1}{3}$. Thus, by Lemma 1 we can use (2), (3), and (4) to compute the local PP of *Q*. By the assumption that (coalescent unit) branch lengths in the species tree are generated by the Yule process, and by Lemma 2, our prior becomes the equation shown in (5). Calculation of (6) follows from manipulating (2), (3), and (4), as detailed in the Supplementary Material online.

#### Examples

**Fig. 2:**
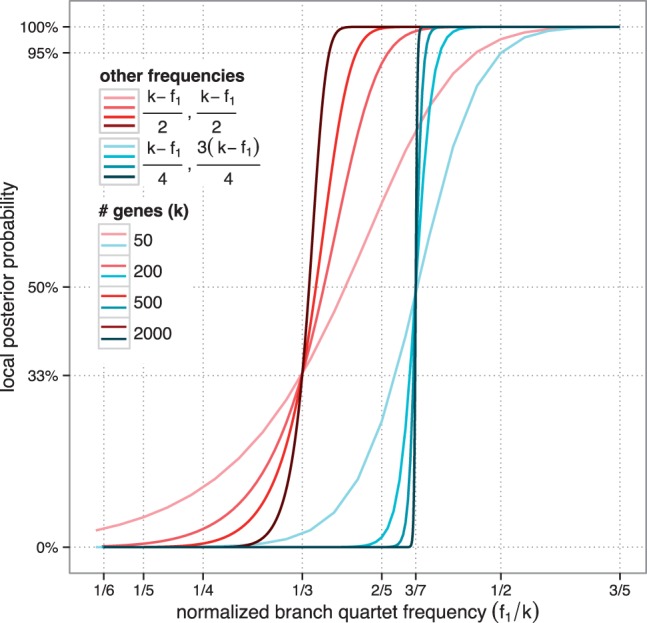
The local PP of a branch as a function of its normalized quartet support for varying numbers of genes. Red lines: alternative topologies have equal frequencies (thus, conform to properties of the MSC for *x* > 1/3); Blue lines: alternative topologies do not have equal frequencies (contrary to the MSC).

The local PP is a function of the number of genes and the quartet frequency of a branch. As figure 2 shows, a branch that appears in 40% of gene trees has a low 66.1% PP if 50 gene trees given (and alternative topologies are equally frequent); however, with 200 or 500 genes, the same branch will have 93.0% or 99.7% PP, respectively. Thus, a high discordance branch where 60% of genes do not agree with the species tree can still be resolved with high confidence given enough genes. Moreover, the PP is affected not just by the frequency of the topology being scored, but also by the frequency of the two alternatives. For example, if our branch of interest appears in 40% of gene trees, but a second alternative appears in three-quarters of the remaining genes (i.e., 45% of genes), the branch with 40% frequency has only a 1.90% PP (fig. 2).

### Calculation of Branch Length

Given the true parameter *θ* for a correct branch, its length in coalescent units (Degnan and Rosenberg 2006) is simply $-\ln\;\frac{3}{2}(1-\theta )$. Thus, we can prove (see Supplementary Material online):

Theorem 2 Under conditions of Theorem 1, and assuming the branch represented by *Q* is in the species tree, the ML estimate for its length is $-\ln\;\frac{3}{2}\left(1-\frac{z_{1}}{n}\right)$ and the MAP estimate is $-\ln\;\frac{3}{2}\left(1-\frac{z_{1}}{n+2\lambda }\right)$ when $z_{1}\geq \frac{n}{3}$ and $z_{1}\geq \frac{n+2\lambda }{3}$, respectively; otherwise, ML=MAP=0.

### Calculation of Quartet Support

We now discuss how $\bar{z}$ defined in (1) can be efficiently computed. Note that in the worst case, there can be $\Theta (l^{4})$ quartets around a single branch. Thus, simply enumerating all $n_{ij}$ values and then getting the average can be very slow.

As noted before, each quartet around *Q* has each of its four leaves drawn from a different cluster of *Q*. Recall also that internal nodes of a tree produce a tripartition, and as Mirarab, Reaz, Bayzid, et al. (2014) pointed out, any selection of two leaves from one side of the tripartition and one leaf from each remaining side gives a quartet tree mapped to that tripartition (fig. 1). Let ψ(Q) and ψ(R) give the set of quartet trees around a quadripartition and mapped to a tripartition, respectively. Any quartet tree around *Q* that is induced by a gene tree will be mapped to two internal nodes in that gene tree (fig. 1). Thus,

(7) $$z_{1}=\frac{1}{2m}\sum_{g=1}^{n}\sum_{u=1}^{l-2}|\psi (R_{u}^{g})\cap \psi (Q)|,$$

where $R_{u}^{g}$ is a tripartition for node *u* in the gene tree *g*, and *m* is the number of quartets around *Q*.

We can efficiently compute the number of quartet topologies around *Q* that appear also in a tripartition R (i.e., $\left|\psi (R_{u}^{g})\cap \psi (Q)\right|$) without computing ψ(.) sets. We define a mapping between clusters around *Q* to clusters in R: map two sister clusters in *Q* to a cluster in R, and map the remaining two clusters in *Q* to the remaining two clusters in R (fig. 1). For example, let Q=AB|CD and R=X|Y|Z; a possible matching is to map *A* and *B* to *Y*, *C* to *X*, and *D* to *Z*. There are 12 such matchings between *Q* and R. For each matching, we can compute the number of quartet trees around *Q* that appear in R by multiplying the sizes of the intersection of pairs of clusters in *Q* and R that are mapped to each other. By enumerating all 12 matching and summing the resulting numbers, we get $\left|\psi (R_{u}^{g})\cap \psi (Q)\right|$. Since finding the intersection of two clusters requires Θ(l), computing (7) would require $O(l^{2}n)$ running time. However, we can do better. Mirarab and Warnow (2015) have introduced a Θ(nl) algorithm to compute a sum similar to (7) for scoring a tripartition, based on a post-order traversal of gene trees (instead of analyzing each $R_{u}^{g}$ separately). This algorithm can be adopted here to compute (7) in Θ(nl) (see supplementary fig. S14, Supplementary Material online for the algorithm). Since scoring a tree requires scoring l−3 branches,

Theorem 3 Computing branch lengths and local posterior probabilities of a tree requires $\Theta (l^{2}n)$ time.

### Other Considerations

We implemented our methods in ASTRAL, using the Colt (Hoschek 2002) package for numerical computations. Handling missing data and unresolved gene trees requires extra care. When gene trees have missing data, to compute (7), instead of setting *m* to the number of quartets around *Q*, we need to set it to the average number of quartets present in gene trees (i.e., $\frac{1}{n}\sum_{1}^{3}z_{j}$). Moreover, missing data can cause some genes to miss *all* quartets around *Q*; to account for this, we allow a different n for each branch, and set it to the number of genes that include at least one of the quartets around *Q*. To handle unresolved gene trees, similar to ASTRAL-II, we need to score the quadripartition against all $\left(\begin{array}{c} d\\ 3 \end{array}\right)$ tripartitions around a polytomy with degree *d*.

## Materials and Methods

### Datasets

We use both simulated and biological datasets.

#### Simulated Data

We use two sets of simulated datasets from previous publications: the 200-taxon dataset (called A-200 here) from Mirarab and Warnow (2015) and an avian dataset with 48 taxa from Mirarab, Bayzid, Boussau, et al. (2014). A-200 enables us to test accuracy under heterogeneous conditions with many species, and the avian dataset is used to compare local posterior against MLBS. For both datasets, gene trees are simulated using the MSC, and their branch lengths are then adjusted to be in substitution units and to deviate from the strict molecular clock. Sequence data are next simulated on the modified gene trees using GTR + Γ, and ML gene trees are estimated from the data. On the avian dataset, bootstrapped gene trees are also available. For both datasets, in addition to true species trees, we have estimated species trees (ASTRAL and NJst on estimated gene trees, and concatenation using ML). We show results for ASTRAL and true species tree here and show the rest in the Supplementary Material online.

##### A-200

The 201-taxon datasets (200 ingroups plus an outgroup, treated like other taxa here) are simulated using SimPhy (Mallo et al. 2015), and has three levels of ILS (table 1), with true discordance that ranges from very low to very high (supplementary fig. S1, Supplementary Material online). Each replicate of the simulation has its own species tree, and the ILS level is controlled by changing the tree length (500k, 2M, and 10M generations). Mirarab and Warnow (2015) generated species trees using the Yule process with two speciation rates (10^−^^6^ and 10^−7^ per generation) for each tree length, but here we combine the two rates into one dataset to get twice the number of replicates. SimPhy automatically introduces deviation from the strict clock by drawing species, gene, and gene/species-specific rate multipliers from predefined distributions. Similarly, the number of sites for each gene is randomly chosen; see Mirarab and Warnow (2015) for details. ML gene trees are estimated using FastTree-II (Price et al. 2010), with a wide range of estimation error (table 1 and supplementary fig. S1, Supplementary Material online). Species trees are estimated based on all 1,000 genes per replicate, or on subsets of 200 or 50 genes. The ASTRAL species trees error for various datasets ranges between average 3% and 19% (table 1).

**Table 1:** Properties of Simulated Datasets.

	**Cond.**	***L***	***n***	**R**	**ILS**	**GE**	**SE**
A-200	Low-ILS	201	1,000	100	15%	25%	6%, 4%, 3%
A-200	Med-ILS	201	1,000	100	34%	31%	9%, 6%, 4%
A-200	High-ILS	201	1,000	100	69%	47%	19%, 10%, 6%
Avian	1,500 bp	48	1,000	20	47%	31%	5%
Avian	1,000 bp	48	1,000	20	47%	39%	6%
Avian	500 bp	48	1,000	20	47%	54%	8%
Avian	250 bp	48	1,000	20	47%	67%	15%

Note.—*l*, number of species; *n*, maximum number of genes (A-200 also has 50 and 200); *R*, number of replicates; *ILS*, average normalized RF (Robinson and Foulds 1981) distance (AD) between the true species tree and true gene trees; *GE*, AD between true and estimated gene trees; *SE*, AD between true and ASTRAL species trees (for A-200, with 50, 200, and 1,000 genes, respectively).

##### Avian

The avian dataset has 48 taxa, and is simulated to emulate the whole-genome dataset of Jarvis et al. (2014), possibly overestimating the true amount of ILS (Gatesy and Springer 2014; Mirarab, Bayzid, Boussau, et al. 2014). Here, we use four conditions, with 20 replicates that each includes 1,000 genes, all simulated based on the same avian-like species tree. Our four conditions differ in terms of the number of sites per gene (250, 500, 1,000, or 1,500 bp), creating varying levels of gene tree estimation error (table 1). ML gene trees are estimated using RAxML (Stamatakis 2014), and 200 replicates of bootstrapping are performed. Average ASTRAL species tree error ranges from 5% to 15%, depending on the gene tree error (table 1). We used site-only MLBS to get BS support values. A single branch in our true tree was extremely short (almost a polytomy, with a length of 10^–^^6^). When discussing branch length accuracy, we ignore that branch; results including that branch will also be shown for completeness.

##### Biological Dataset

We reanalyze four published datasets: a 103-taxon 424 gene plant dataset by Wickett et al. (2014), a 46-taxon 310 gene angiosperm dataset by Xi et al. (2014), a 48-taxon 2022 (binned) supergene tree dataset by Jarvis et al. (2014), and a 201-taxon 256 gene avian dataset by Prum et al. (2015).

### Evaluation Procedure

We study three questions in our evaluation:

1. How accurate are branch lengths and support values when assumptions of our model are met?
2. How do violations of the model assumptions impact the result?
3. How do local posterior probabilities compare to site-only MLBS?

To answer these questions, we use both true gene trees and estimated gene trees to score both true and estimated species trees. For every internal branch in the species tree being scored, we also score its two alternative topologies. For example, if branch AB|CD (fig. 1) appears in the species tree, we also score AD|BC and AC|BD. In our estimations, we use the Yule prior with fixed $\lambda =\frac{1}{2}$, but note that the true *λ* in our A-200 simulations ranges from 0.06 to 1.19.

With true species trees and true gene trees, all model assumptions are met. When estimated gene trees are used instead of true gene trees, we violate the assumption that input gene trees follow properties of the MSC model. When estimated species trees are scored, the locality assumption is potentially violated (i.e., each of the four clusters around a branch may be incorrect).

## Measurement

### 

#### Posterior

Despite the long-standing debate about correct interpretations of various measures of support (e.g., Felsenstein and Kishino 1993; Hillis and Bull 1993; Susko, 2009; Salichos and Rokas 2013), biologists typically use support to judge branch reliability. A common practice is to ignore branches below a certain threshold of support and only interpret the remaining branches as biologically meaningful (0.95 for posterior and 70% for bootstrap are often used). Our evaluation procedure takes a similar approach; we use varying thresholds of support and count the number of true and false branches with support at least equal to the threshold. For a threshold *s*, the measures we use are precision (the percentage of branches with support ≥s that are correct), recall (the percentage of all true branches that have support ≥s), and false positive rate (FPR) (the percentage of all false branches that have support ≥s). We also draw the ROC curve (i.e., recall versus FPR).

MLBS and posteriors values are not directly comparable. Therefore, it is pointless to compare the precision or recall of MLBS and posterior for a given threshold. Instead, we use the ROC curve, which is agnostic to the exact interpretation of the threshold; it simply shows which method results in a better trade-off between false negative and false positive branches. Moreover, comparing to MLBS was only feasible on the avian dataset, where gene bootstrapping was doable. On the A-200 dataset (300 replicates each with 1,000 genes of 201 taxa) bootstrapping was not computationally feasible.

#### Branch Length

We measure branch length accuracy by comparing true and estimated lengths for each branch. Since this can be done only for correct branches, we measure branch length accuracy for the true species tree topology. Given *b* branches, and letting *w_i_* and ${\hat{w}}_{i}$ indicate the true and estimated branch lengths, we use the logarithmic (log) error defined as $\frac{1}{b}\sum_{1}^{b}|{\log\;}_{10}(w_{i})-{\log\;}_{10}({\hat{w}}_{i})|$. We also plot log of estimated versus true values. In addition, we show the root mean squared error, defined as $\sqrt{\frac{1}{b}\sum_{1}^{b}{\left(w_{i}-{\hat{w}}_{i}\right)}^{2}|}$. On Avian datasets, we compare the error of MP-EST and ASTRAL.

## Results

### A-200 Dataset

#### Posterior

##### True Trees

When true species trees are scored with true gene trees, the precision of branches with 0.99 pp or higher is 100% for all model conditions, and is at least 99.8% for the 0.95 threshold (table 2). Thus, there are very few false positive branches that have high local pp, a trend that continues if we further lower the threshold to 0.9 (supplementary fig. S2, Supplementary Material online). With the 0.95 threshold, the percentage of true branches that are recovered (recall) ranges from very high (98.7%) for the model condition with low ILS and 1,000 gene trees to moderate (55.0%) for the most challenging dataset with high ILS and only 50 genes (table 2). As desired, increasing ILS and reducing the number of genes both reduce the recall while maintaining high precision (supplementary fig. S2, Supplementary Material online).

**Table 2:** Precision (and Recall) of Local Posterior Probabilities on A-200 Dataset.

	n	**True species tree**				**ASTRAL species tree**			
		**True gene tree**		**Estimated gene tree**		**True gene tree**		**Estimated gene tree**	
		**0.99**	**0.95**	**0.99**	**0.95**	**0.99**	**0.95**	**0.99**	**0.95**
Low ILS	1,000	100.0 (98.3)	100.0 (98.7)	98.6 (94.6)	98.4 (95.4)	98.8 (98.4)	98.8 (98.8)	98.0 (95.1)	97.8 (95.9)
Low ILS	200	100.0 (95.9)	100.0 (96.8)	99.1 (90.2)	98.9 (91.9)	98.9 (96.2)	98.8 (97.1)	98.7 (90.6)	98.5 (92.4)
Low ILS	50	100.0 (91.1)	100.0 (93.2)	99.6 (81.0)	99.3 (85.0)	98.7 (91.6)	98.6 (93.6)	99.4 (81.6)	99.0 (85.6)
Med ILS	1,000	100.0 (95.1)	100.0 (96.2)	98.9 (90.8)	98.7 (92.4)	99.3 (95.4)	99.2 (96.6)	98.6 (91.2)	98.4 (92.8)
Med ILS	200	100.0 (89.2)	100.0 (91.3)	99.4 (82.6)	99.2 (85.7)	99.4 (90.0)	99.4 (92.1)	99.3 (83.3)	99.0 (86.5)
Med ILS	50	100.0 (79.0)	99.9 (83.2)	99.7 (70.0)	99.5 (75.2)	99.4 (81.0)	99.2 (85.2)	99.7 (71.3)	99.5 (76.6)
High ILS	1,000	100.0 (83.0)	100.0 (86.0)	99.4 (77.5)	99.2 (81.3)	99.7 (84.2)	99.6 (87.1)	99.4 (78.3)	99.2 (82.1)
High ILS	200	100.0 (67.3)	100.0 (72.8)	99.8 (60.3)	99.6 (66.6)	99.8 (69.6)	99.7 (75.1)	99.8 (61.9)	99.6 (68.3)
High ILS	50	100.0 (46.0)	99.8 (55.0)	99.8 (38.6)	99.6 (47.7)	99.8 (50.0)	99.4 (59.7)	99.8 (41.1)	99.6 (50.6)

Note.—For local PP thresholds 0.95 and 0.99, we show the precision and recall (shown parenthetically) when true or ASTRAL species trees are scored with true or estimated gene trees.

##### Estimated Gene Trees

When true species trees are scored on estimated gene trees instead of true gene trees, precision slightly drops from 100% to between 98.4% and 99.8%. The recall, however, is impacted more and is reduced by as much as 10% (table 2 and supplementary fig. S2, Supplementary Material online). Thus, gene tree estimation error has a small impact on the precision but a substantial impact on the recall.

**Fig. 3:**
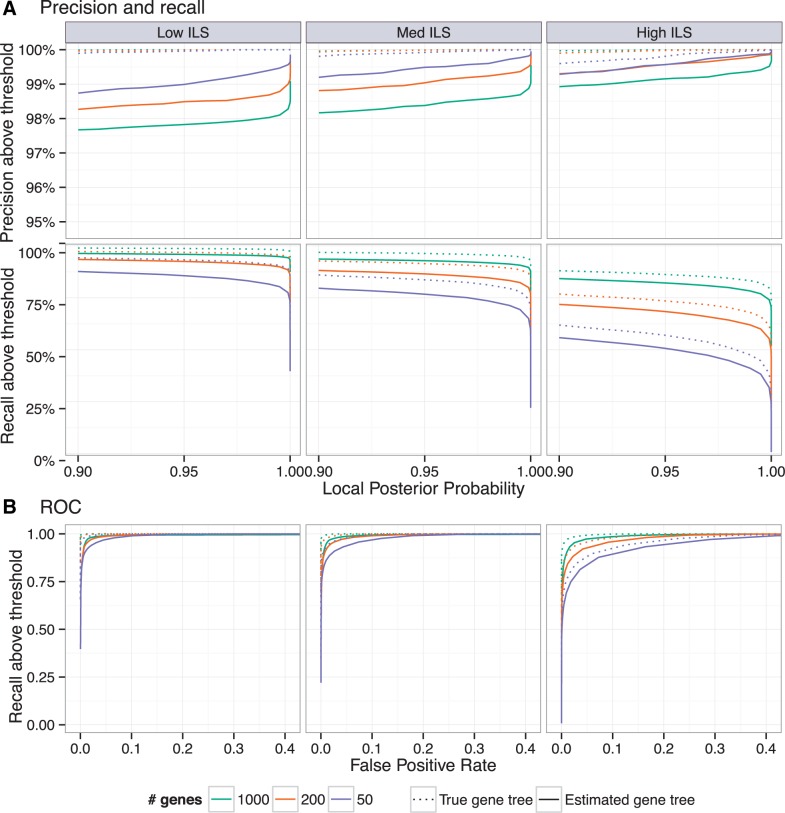
Evaluation of local PP on the A-200 dataset with ASTRAL species trees. See supplementary figures S2–S4, Supplementary Material online for other species trees. (*A*) Precision and recall of branches with local PP above a threshold ranging from 0.9 to 1.0 using estimated gene trees (solid) or true gene trees (dotted). (*B*) ROC curve (recall vs. FPR) for varying thresholds (figure trimmed at 0.4 FPR). Columns show different levels of ILS.

The impact of the threshold is also interesting. Going from the 0.99–0.95 threshold, as expected, recall improves (e.g., from 39% to 48% for high ILS, 50 genes) but reductions in the precision are very small (at most 0.3%). Thus, a 95% threshold results in meaningful improvements in the recall without substantially sacrificing the precision. The ROC curves (fig. 3*B* and supplementary S2, Supplementary Material online) further explore the tradeoff between increasing recall and allowing false positive branches.

##### Estimated Species Trees

By scoring estimated species trees we study the impact of violating the locality assumption. We show results for ASTRAL here, but similar results are obtained with NJst and concatenation (supplementary figs. S2 and S4, Supplementary Material online).

Precision and recall are remarkably similar between ASTRAL and true species trees, especially with estimated gene trees (table 2). Comparing true species trees and ASTRAL on estimated gene trees, the precision is reduced at most by 0.6% while the recall is surprisingly increased, by up to 2.9%. The impact of violating locality assumptions is more pronounced when true gene trees are used. Once again, precision is reduced (by as much as 1.4%) and the recall is increased (up to 4.7%). Thus, moderate violations of the locality assumption have minimal impact.

We note that in our analyses, deviations from the locality assumption are moderate but realistic, as most ASTRAL trees have a relatively high accuracy (table 1). The least accurate ASTRAL trees have 19% RF distance to the true species tree (50 genes and high ILS), which means 81% of the clusters in the estimated tree remain correct. Nevertheless, it is interesting that violating the locality assumption for up to 19% of clusters has minimal impact on the precision and positive impact on the recall.

#### Branch Length

**Fig. 4:**
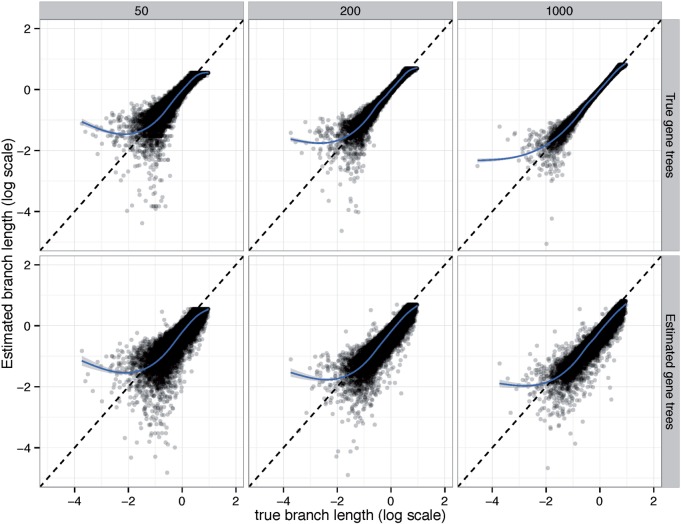
Branch length accuracy on the A-200 dataset with Medium ILS. See supplementary figures S7 and S8, Supplementary Material online for low and high ILS. The estimated branch length is plotted against the true branch length in log scale (base 10). Blue line: a fitted generalized additive model with smoothing (Wood 2011).

The accuracy of branch lengths is dramatically impacted by gene tree estimation error, the number of genes, and the amount of ILS (table 3). With 1,000 true gene trees, the logarithmic error is very low, ranging from 0.03 to 0.10 (which correspond to branches that on average are, respectively, 7% or 25% shorter or longer than true branches). As the number of genes is reduced, the logarithmic error predictably goes up, but with true gene trees, it never exceeds 0.25. Moreover, with true gene trees, the error is largely unbiased, except perhaps for very short or long branches that are hard to estimate correctly with a limited number of genes (fig. 4 and supplementary figs. S7 and S8, Supplementary Material online).

**Table 3:** Branch Length Accuracy on the A-200 Dataset.

**Dataset**	***n***	**Log Err**		**RMSE**	
		**True gt**	**Est. gt**	**True gt**	**Est. gt**
Low ILS	1,000	0.10	0.42	5.57	6.75
Low ILS	200	0.16	0.44	6.22	6.99
Low ILS	50	0.25	0.48	6.84	7.29
Med ILS	1,000	0.03	0.20	0.22	0.86
Med ILS	200	0.07	0.22	0.44	0.91
Med ILS	50	0.13	0.26	0.74	1.05
High ILS	1,000	0.06	0.15	0.03	0.13
High ILS	200	0.11	0.18	0.07	0.15
High ILS	50	0.18	0.24	0.14	0.19

Note.—Logarithmic error (Log Err) and RMSE are shown for true species trees scored with true gene trees or estimated gene trees (Est. gt).

Branch length error dramatically increases when estimated gene trees are used. Low ILS conditions are impacted the most by gene tree error (table 3 and supplementary fig. S7, Supplementary Material online). For example, with 1,000 estimated gene trees and low ILS, log error is 0.42, corresponding to estimated branches that are on average 2.6 times too short or long. Moreover, the error is biased towards underestimation, especially for low ILS (fig. 4, and supplementary figs. S7 and S8, Supplementary Material online). This pattern is not surprising because as we will show, gene tree error tends to increase observed gene tree discordance and branch lengths are a function of observed discordance.

### Avian

On the avian dataset, we compare local posterior probabilities against branch support generated using site-only MLBS with estimated gene trees and ASTRAL species trees. Here, we also study the impact of increasing levels of gene tree estimation error by decreasing the number of sites per gene from 1,500 bp to 250 bp.

### Posterior and MLBS

The precision of local PP is 100% for the 0.99 threshold, regardless of the numbers of sites, but the recall ranges from 81% for the 1,500 bp model condition to 69% for 250 bp (supplementary table S1 and figs. S5 and S6, Supplementary Material online). Precision is at least 99.8% for the 0.95 threshold, and the recall is between 71.5% and 84.7%, depending on the model condition (an improvement of 2–5% compared with the 0.99 threshold). Lowering the support threshold all the way to 0.7 still retains at least 99.1% accuracy and increases the recall to between 78.3% and 91.4%. Therefore, the local posterior probabilities allow very few false positives with high support but also miss some true positives (and thus may be conservative).

**Fig. 5:**
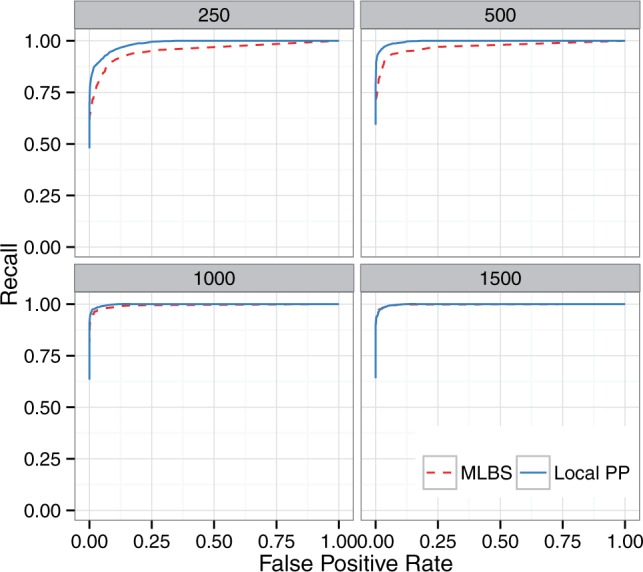
ROC curve for the avian dataset based on MLBS and local PPPP support values. Boxes show different numbers of sites per gene (controlling gene tree estimation error).

Nevertheless, local posterior probabilities are less conservative than MLBS support values and have better recall. As the ROC curves show (fig. 5), for the same number of false positives branches, local posterior probabilities result in better recall than MLBS. This pattern is more pronounced for shorter alignments, which have increased gene tree error. For example, for the 250 bp model condition, if we choose a support threshold that results in 0.01 FPR, with local posterior values, we still recover 84% of correct branches, whereas with MLBS, the same FPR results in retaining 70% of correct branches. Thus, for a desired level of precision, better recall can be obtained using local posterior probabilities.

#### Branch Length

**Fig. 6:**
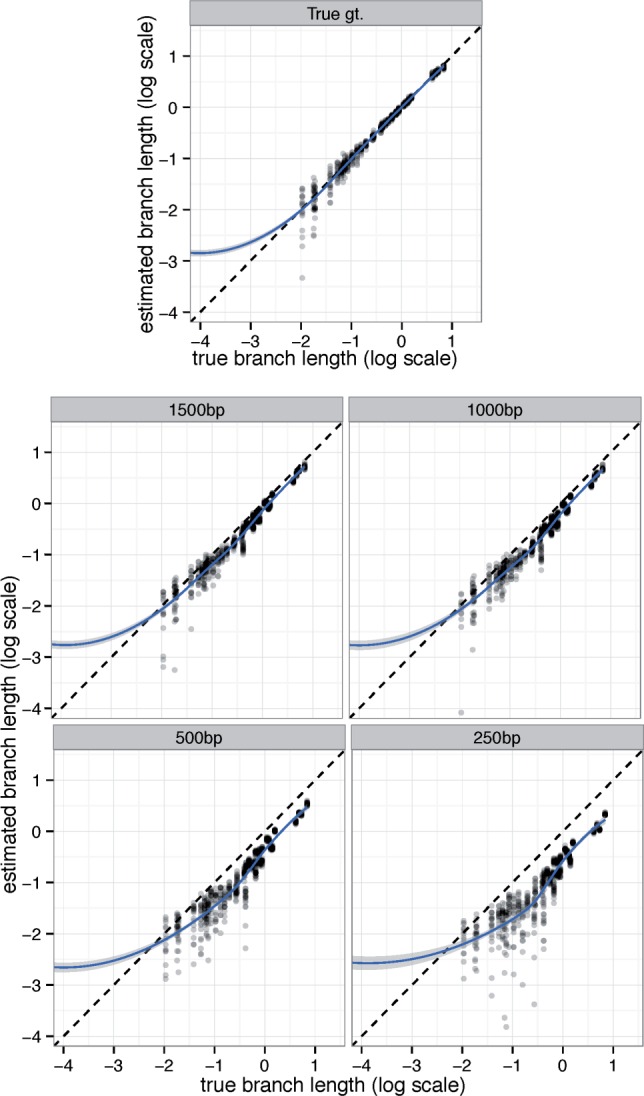
ASTRAL branch length accuracy on the avian dataset. Log transformed estimated branch lengths are shown versus true branch lengths, and a generalized additive model is fitted to the data. One branch with length 10^−6^ is trimmed out here, but full results, including MP-EST, is shown in supplementary figure S9, Supplementary Material online.

Branch length accuracy on the avian dataset was a function of gene tree estimation error whether ASTRAL or MP-EST was used (table 4). With true gene trees, branch length log error was only 0.06, corresponding to branches that are about 14% shorter or longer than the true branch. As gene tree estimation error increases with reduced number of sites (see table 1 for gene tree error statistics), the branch length error also increases. Thus, while 1,500 bp genes give 0.17 log error, 250 bp genes result in 0.59 error, which corresponds to branches that are on average 3.9 times too short or long. Moreover, unlike true gene trees, the error in branch lengths estimated based on estimated gene trees is biased toward underestimation (fig. 6), a pattern that increases in intensity with shorter alignments.

**Table 4:** Branch Length Accuracy for the Avian Dataset.

**No. of sites**	**Log Err**		**RMSE**	
	**ASTRAL**	**MPEST**	**ASTRAL**	**MPEST**
True gt.	0.06 (0.10)	0.07 (0.11)	0.44 (0.44)	0.30 (0.30)
1,500	0.17 (0.20)	0.14 (0.18)	0.83 (0.83)	0.70 (0.70)
1,000	0.22 (0.27)	0.22 (0.25)	1.08 (1.07)	1.01 (1.00)
500	0.37 (0.42)	0.42 (0.46)	1.65 (1.64)	1.65 (1.64)
250	0.59 (0.63)	0.81 (0.84)	2.25 (2.24)	2.28 (2.26)

Note.—Logarithmic error and root mean squared error are shown for true species trees scored with true gene trees or estimated gene trees with various numbers of sites using ASTRAL and MP-EST. An extremely short branch with length 10^−6^ was removed from the calculations, but error including that branch is shown parenthetically.

ASTRAL and MP-EST have similar branch length accuracy measured by log error for highly accurate gene trees, but ASTRAL has an advantage with increased gene tree error (supplementary fig. S10, Supplementary Material online and table 4). Error measured by root mean squared error (RMSE) (which emphasizes the accuracy of long branches) is comparable for the two methods, but MP-EST has a slight advantage given accurate gene trees.

### Biological Datasets

For each biological dataset, we show MLBS support and the local posterior probabilities, computed based on RAxML gene trees available from respective publications. We also collapse gene tree branches with <33% bootstrap support and use these collapsed gene trees to draw local posterior probabilities. For ease of discussion, we show local posterior probabilities as percentages and refer to them simply as posterior or collapsed posterior (for values based on collapsed gene trees). We discuss the confidence in important branches in each tree.

#### 

##### 1KP

**Fig. 7:**
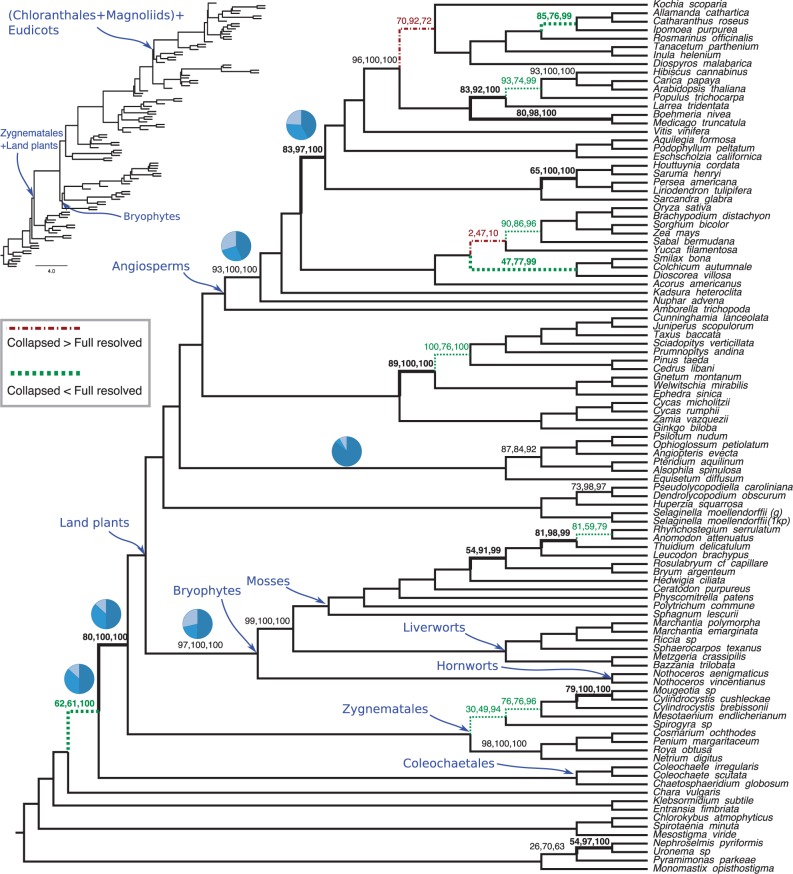
ASTRAL tree on the 1KP dataset of Wickett et al. (2014) (103 taxa and 424 genes). On each branch, three support values are shown: BS (using site-only MLBS), local posterior computed on fully resolved ML gene trees, and local posterior computed on collapsed ML gene trees (removing branches with <33% BS). Branches with no designation have 100% support with all three measures. Dotted/green lines (dashed/red lines): collapsing low support gene trees branches increases (decreases) posterior by at least 10%. Bold: collapsed posterior is at least 10% higher than BS. Inset: ASTRAL tree with branch lengths in coalescent units using collapsed genes (terminals lengths drawn arbitrarily). Pie-charts (for selected edges): relative frequencies of the three quartet topologies around a branch in collapsed gene trees.

Three of the key relationships studied by Wickett et al. (2014) are the sister branch to land plants, the base of the angiosperms, and the relationship among Bryophytes (hornworts, liverworts, and mosses). In the ASTRAL tree, many branches have full support regardless of the measure of the support used, but the remaining branches reveal interesting patterns (fig. 7). The sister relationship between Zygnematales and land plants receives a moderate 80% BS, but has 100% posterior. Wickett et al. (2014) also recovered this relationship by concatenation of various data partitions. There are 12 other branches that have collapsed posteriors that are at least 10% higher than BS (fig. 7); no branch has substantially higher BS than collapsed posterior. Collapsed posterior for monophyly of Bryophytes and for Amborella as sister to other angiosperms are 100% (compared with 97% and 93% BS, respectively).

When we collapse low-support branches in gene trees, posterior goes up for several branches: nine branches have improvements of 10% or more, and only two branches have comparable reductions. An interesting case is Coleochaetales as sister to Zygnematales + land plants, which has only 62% BS and 61% posterior, but has 100% collapsed posterior. Finally, note that several branches have low posterior, even after collapsing.

Our estimated branch lengths are short for several nodes. For example, the branch that unites (Chloranthales +Magnoliids) and Eudicots has a length of 0.14 in coalescent units. Other branches that have been historically hard to recover also tend to have short branches; however, these are not necessarily extremely short branches that would implicate anomaly zone [two adjacent branches below 0.1 will result in an anomaly zone (Rosenberg 2013)]. For example, Bryophytes had a length of 0.29, and Zygnematales + land plants had a length of 0.28. These values, while short, are not below the often-cited 0.1 threshold. Moreover, as our simulation study showed, we caution that branch lengths tend to be underestimated because of gene tree error and these numbers should be treated as lower bounds.

##### Angiosperms

Xi et al. (2014) have used 310 genes to study the base of the angiosperm tree, a question of intense debate (e.g., Soltis et al. 2011; Zhang et al. 2012; Goremykin et al. 2013; Simmons and Gatesy 2015). Unlike the MP-EST tree by Xi et al. (2014), but similar to concatenation on this dataset and ASTRAL and concatenation on 1KP, the ASTRAL tree recovers Amborella as sister to the rest of the angiosperms. This relationship has 75% BS, but it’s posterior and collapsed posterior are 100% (supplementary fig. S11, Supplementary Material online). The length of this branch is estimated to be 0.160, almost exactly matching the length estimated on the 1KP dataset (0.156).

##### Avian (Genomes)

Jarvis et al. (2014) used whole-genomes of 48 bird species to resolve long-standing questions about relationships at the base of Neoaves. We reanalyzed their 2,022 supergene trees (binned gene trees; see Mirarab, Bayzid, Boussau, et al. 2014) using ASTRAL, which produced a tree with a wall of short branches at the base of Neoaves (supplementary fig. S12, Supplementary Material online); 12 branches are below 0.1 coalescent units, and another 11 are below 0.5. However, when low-support branches in gene trees were collapsed, branch lengths increase by a median of 0.23 units. Nevertheless, 11 branches remain below 0.1, and another four branches are below 0.5. Our results support the hypothesis that avian big bang. Feduccia (2003) gave rise to very short branches, but quantifying the exact lengths remains difficult because of gene tree error.

Support values on the avian tree also revealed interesting patterns. Despite the large number of supergene trees (2,022), which should increase support (fig. 2), several key branches have low posterior. For example, the position of Hoatzin (arguably, the most difficult avian order to place) and the two branches around it have collapsed posterior below 50% and posterior below 75% (supplementary fig. S12, Supplementary Material online). Similarly, at the base of Neoaves, a clade containing land birds, water birds, and Caprimulgiformes has full support, but the sister to this large group has only 61% posterior and 80% collapsed posterior. However, other challenging relationships have full support (e.g., falcons as sister to parrots + passerines, and seriema as sister to this group, or a clade containing owls, eagles, and vultures). Thus, despite large number of input trees, posterior probabilities reveal some uncertainty. Finally, on this dataset, unlike the 1KP dataset, four branches have substantially lower posterior compared with BS, and posteriors are higher than collapsed posteriors in some cases.

##### Avian (High Sampling)

Prum et al. (2015) used their dataset of 259 genes and 201 species to study the avian tree with high taxon sampling. The ASTRAL tree reported by Prum et al. (2015) has low MLBS (supplementary fig. S13, Supplementary Material online), and many branches remain poorly supported with posterior probabilities (the median difference between BS and collapsed posterior was 0). Moreover, many of the most interesting relationships are poorly supported. For example, the sister to parrots + passerine has only 30% MLBS support, 83% local posterior support, and 0% collapsed posterior. These low support values are encouraging because the sister to parrots + passerine is likely recovered incorrectly in this tree, as most recent studies put falcons as sister to this group (Suh et al. 2011; Kimball et al. 2013; McCormack et al. 2013; Jarvis et al. 2014). Overall, despite its large taxon sampling, this dataset provides little resolution for the early Neoaves radiation using ASTRAL because of the insufficient gene count for this high level of ILS. Just like the avian genomic data, here we obtain a wall of short branches around the assumed rapid radiation of Neoaves (supplementary fig S13, Supplementary Material online).

## Discussions

The local posterior probabilities introduced in this article can be computed quickly and without a need for extensive MCMC sampling or bootstrapping. Computing posteriors for a species tree with 200 taxa and 1,000 genes takes only 10 s and for a dataset with 1,000 taxa and 1,000 genes, takes about 3 min on a laptop machine. This extremely fast computation is possible only because of the two main assumptions of the method: that true MSC-generated gene trees are given and the locality assumption (i.e., the four clusters around each internal branch are present in the true tree). These assumptions can both be violated on real data. Recognizing this fact, our simulations include conditions that violate these two assumptions by introducing plenty of gene tree estimation error (ranging from average RF distance of 25–67%) as well as species tree error (table 1 and supplementary fig. S1, Supplementary Material online).

Our method allows very few false positives with high support, a pattern that is retained even with high levels of gene tree estimation error. It could be argued that our method is perhaps too conservative and underestimates support. Nevertheless, local posterior probabilities were *less* conservative than MLBS, the only viable alternative for large datasets. Despite allowing very few false positives with high support, the method generally had high recall (i.e., true branches with high support) except for very few genes for a given amount of ILS. Reassuringly, increased gene tree estimation error only negatively impacted recall but retained very high precision. While underestimation of support is not desirable, the abundance of false branches with high support would be a more serious problem.

A practical question is at what threshold of support a branch can be judged reliable. The answer depends on factors such as the FPR desired and the amount of gene tree error. Nevertheless, it seems that the commonly used 0.95 threshold results in very high precision while retaining moderately high recall. In our analyses, even lower thresholds (e.g., 0.9 or even 0.7) give high precision, while increasing the recall.

### Gene Tree Estimation Error

An interesting pattern was that with estimated gene trees (but not with true gene trees), at a given threshold, support values are more precise for high ILS compared to low ILS (fig. 3*A*). We postulate that this effect is related to the larger impact that gene tree estimation error has on the total amount of observed discordance for low ILS compared with high ILS conditions. Consistent with this explanation, we also observed a larger degradation of branch length accuracy in going from true to estimated gene trees for low ILS conditions compared with high ILS (table 3).

**Fig. 8:**
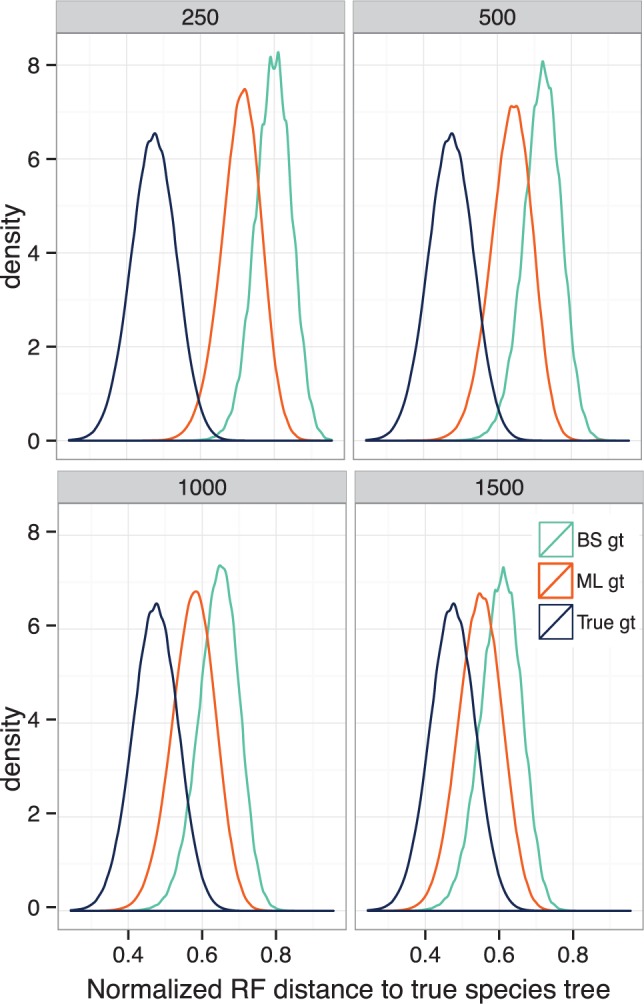
Gene tree discordance for the avian dataset. We show the density plot of the normalized RF distance between the true species tree and true gene trees, ML gene trees, and BS gene trees for four different model conditions.

The issue of gene tree estimation error is at the heart of why we saw a need for developing this new method. Sets of site-resampled bootstrap gene trees tend to have increased levels of discordance with regard to the species tree (and also among themselves) compared with ML gene trees, especially when each gene has a limited phylogenetic signal. Bootstrapped gene trees have much higher rates of discordance than either true gene trees or ML gene trees (fig. 8). It is expected that bootstrapped replicates of a dataset result in noisier estimates of parameters than ML; however, the added error by bootstrapping should not be *biased*. For MLBS, the input to the summary method is not just a noisy dataset, but a *biased* one with increased levels of discordance. We postulate this bias is the reason for the underperformance of MLBS.

Our method, on the other hand, does not require bootstrapping and uses the best available gene trees (e.g., estimated ML gene trees). While ML gene trees are still biased toward increased discordance (and hence reduced branch length), they are better than bootstrapped gene trees (fig 8). The downside of our approach is that gene tree uncertainty is not considered directly. Thus, it was reassuring to see that our method remains precise with high gene tree estimation error. To account for gene tree estimation error, one can collapse the most poorly resolved branches in gene trees when computing support. As we show in our analyses of biological data, this practice seems promising. However, we note that when collapsing branches, one should be careful not to introduce bias, which can happen with aggressive filtering. We choose to collapse branches with support below 33% (which can be considered randomly resolved). Future work needs to further study the effect of collapsing low-support branches in gene trees on both branch length and support.

The impact of gene tree estimation error was most clear with estimates of the branch length. The branch lengths produced by our method showed encouraging patterns (e.g., consistency across biological datasets); nevertheless, our estimated branch lengths are not immune to underestimation that is seen often with other summary methods. Thus, we suggest branch lengths from ASTRAL and other summary methods should be interpreted with care in the presence of gene tree estimation error.

### High Support Despite High Discordance

An important observation, predicted by the theory but sometimes lost in the scientific debate about discordance, is that high confidence for a correctly inferred relationship can emerge even with high levels of discordance. As figure 2 shows, a branch that appears in only 40% of gene trees can still be resolved with high confidence if a sufficient number of genes are available (e.g., ∼500). For example, in the 1KP tree, the branch that puts Zygnematales as sister to land plants appeared in only 49% of collapsed gene tree quartets and the branch making Bryophytes monophyletic only appeared in 50% of them; both branches, however, have a posterior of 1.0. We have implemented an option in ASTRAL to output the percentage of gene tree quartets that agree with each of the three resolutions around a branch. Pie charts in figure 7 give examples of these relative quartet frequencies.

A question that biologists often face is the number of genes required to resolve a branch. The number of genes required to obtain high-resolution and low-FPRs depends on the model condition. With higher ILS, more genes are required, an observation that is not surprising. However, our method can be extended to estimate the number of genes that might be required to resolve a tree (with an estimated level of ILS).

### Limitations and Future Work

Promising approaches for incorporating gene tree uncertainty into local posterior probabilities exist. For example, one can weight each gene tree quartet around a branch by its SH-like support, BS, or advanced measures like concordance (Ané et al. 2007). Moreover, comparing our method against Bayesian co-estimation methods on small datasets where they can run will be interesting. Furthermore, we did not investigate the impact of changing prior parameters (*λ*); nor did we explore other prior functions, such as Dirichlet distributions (conjugate to multinomial) or birth–death processes. We leave these for future work.

We violated some but not all assumptions of our method in the experimental results. The sequence evolution models used for simulation and inference were both GTR + Γ, but on real data, model violations (e.g., compositional bias) can lead to biased estimates of gene trees. Finally, all our simulated datasets had discordance that was generated only by ILS and estimation error, and not other sources of true biological discordance, such as undetected paralogy or horizontal gene transfer. Future work should further examine the impact of other biological sources of discordance on the reliability of local posterior probabilities.

## Supplementary Material

Supplementary table S1 and figures S1–S15 are available at *Molecular Biology and Evolution* online (http://www.mbe.oxfordjournals.org/).

Supplementary Data
