# Supplementary material for: Fast Coalescent-Based Computation of Local Branch Support from Quartet Frequencies
Source: Mol Biol Evol. 2016 Apr 15;33(7):1654–68. doi: 10.1093/molbev/msw079 (PMC4915361; doi:10.1093/molbev/msw079)
Supplement: Supplementary Data [file supp_33_7_1654__index.html]

Fast Coalescent-Based Computation of Local Branch Support from Quartet Frequencies — Fast Coalescent-Based Computation of Local Branch Support from Quartet Frequencies — Supplementary Data 

# Fast Coalescent-Based Computation of Local Branch Support from Quartet Frequencies

## Supplementary Data

files

- Supplementary Data - pdf file
