# Supplementary material for: Fast Coalescent-Based Computation of Local Branch Support from Quartet Frequencies
Source: Mol Biol Evol. 2016 Apr 15;33(7):1654–68. doi: 10.1093/molbev/msw079 (PMC4915361; doi:10.1093/molbev/msw079)
Supplement: Supplementary Data [file supp_msw079_supplementary-revision.pdf]

Supplementary Material for "Fast coalescent-based computation of local branch support from quartet frequencies"

Erfan Sayyari[1] and Siavash Mirarab*[1]

[1]University of California, San Diego, Department of Electrical and Computer Engineering

Contents

1 Supplementary Figures and Tables **2**

2 Supplementary methods **17**

3 Proofs **17**
- 3.1 Proof of Lemma 1 . . . 17
- 3.2 Proof of Theorem 1 . . . 17
- 3.3 Proof of Theorem 2 . . . 19
- 3.4 Proof of Lemma 2 . . . 20

4 Commands and version numbers **20**
- 4.1 ASTRAL . . . 20
- 4.2 MP-EST . . . 21

List of Figures

- S1 Properties of the A-200 dataset. . . . 3
- S2 Accuracy of local posterior probability on the A-200 dataset with true species trees. 4
- S3 Accuracy of local posterior probability on the A-200 dataset with NJST species trees. 5
- S4 Accuracy of local posterior probability on the A-200 dataset with concatenation. . . 6
- S5 Precision of local posterior probability on the Avian dataset with ASTRAL. . . . 7
- S6 Recall of local posterior probability on the Avian dataset with ASTRAL. . . . 8
- S7 Branch length accuracy on the A-200 Low ILS dataset. . . . 9
- S8 Branch length accuracy on the A-200 High ILS dataset. . . . 9
- S9 Branch length accuracy on the Avian dataset. . . . 10
- S10 Summarized branch length accuracy on the Avian dataset. . . . 11
- S11 Support on the angiosperm dataset . . . 12
- S12 Support on the avian dataset . . . 13
- S13 Support on the avian dataset of Prum et al. . . . 14
- S14 Frequency calculation algorithm. . . . 15
- S15 Topologies of different quartets around a branch might heavily depend on each other 16

List of Tables

- S1 Support accuracy on the avian dataset. . . . 2

*Corresponding author: smirarab@ucsd.edu

1 Supplementary Figures and Tables

Table S1: **Support accuracy on the avian dataset.** Accuracy (and recall) of BS and local posterior probability is show for three thresholds: 0.7, 0.95, and 0.99.

	BS			Local PP with Estimated gene trees			Local PP with True gene trees		
sites	0.70	0.95	0.99	0.70	0.95	0.99	0.70	0.95	0.99
1500	99.4(93.5)	100.0(89.6)	100.0(85.9)	99.9(90.7)	100.0(84.7)	100.0(81.3)	99.9(93.6)	100.0(90.2)	100.0(86.2)
1000	99.6(89.0)	100.0(79.7)	100.0(74.3)	100.0(91.4)	100.0(84.1)	100.0(80.1)	100.0(94.5)	100.0(91.4)	100.0(87.7)
500	98.0(75.7)	99.8(69.8)	100.0(66.8)	99.5(87.7)	99.8(79.8)	100.0(74.4)	99.0(97.4)	99.4(93.8)	99.5(90.6)
250	95.9(71.7)	99.6(63.2)	100.0(57.4)	99.1(78.3)	100.0(71.5)	100.0(69.2)	97.1(97.7)	98.6(94.7)	99.2(92.6)

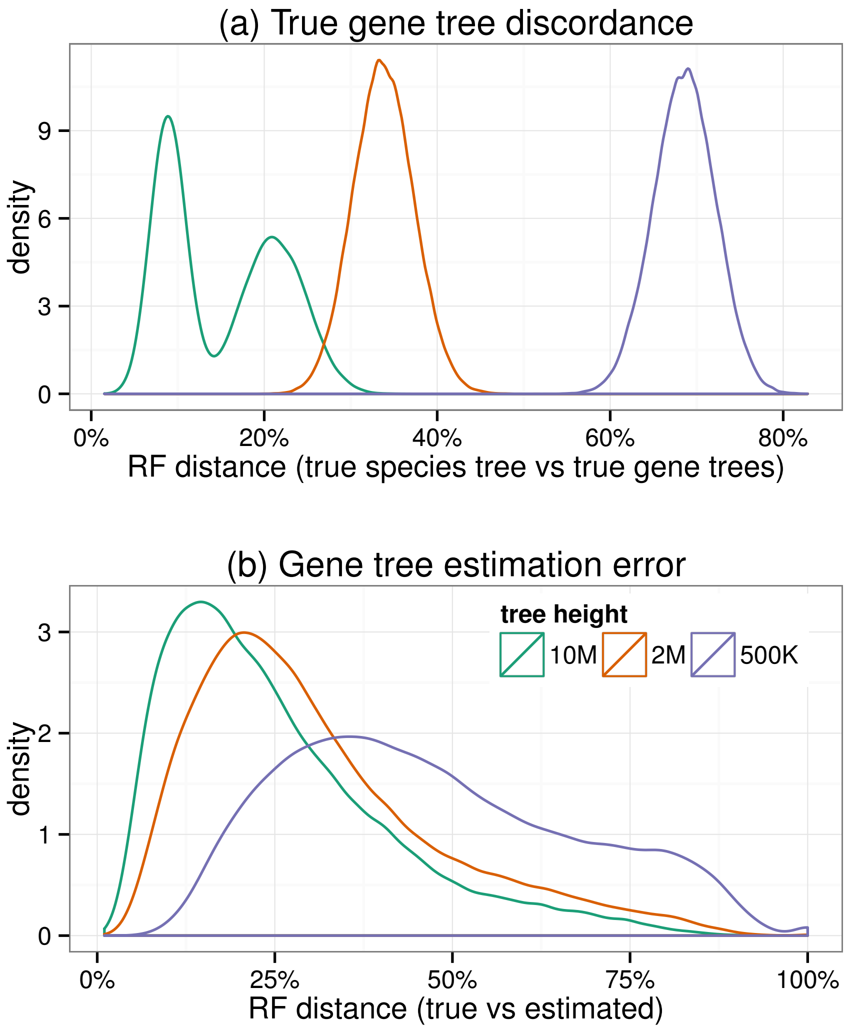

Figure S1: **Properties of the A-200 dataset.** (a) True gene tree discordance is shown, measured as the RF distance (Robinson and Foulds, 1981) between the true species tree and the true gene trees; Three levels of ILS are created by changing tree length (10M, 2M, or 500K generations), resulting in low, medium, and high discordance. The double pick is due to the fact that in Mirarab and Warnow (2015) two different speciation rates are used, but here, we combine both speciation rates into one larger dataset. Bottom: Gene tree estimation error, measured as the RF distance between the true gene tree and the estimated gene tree. Note that the datasets with higher ILS also tend to have higher gene tree error.

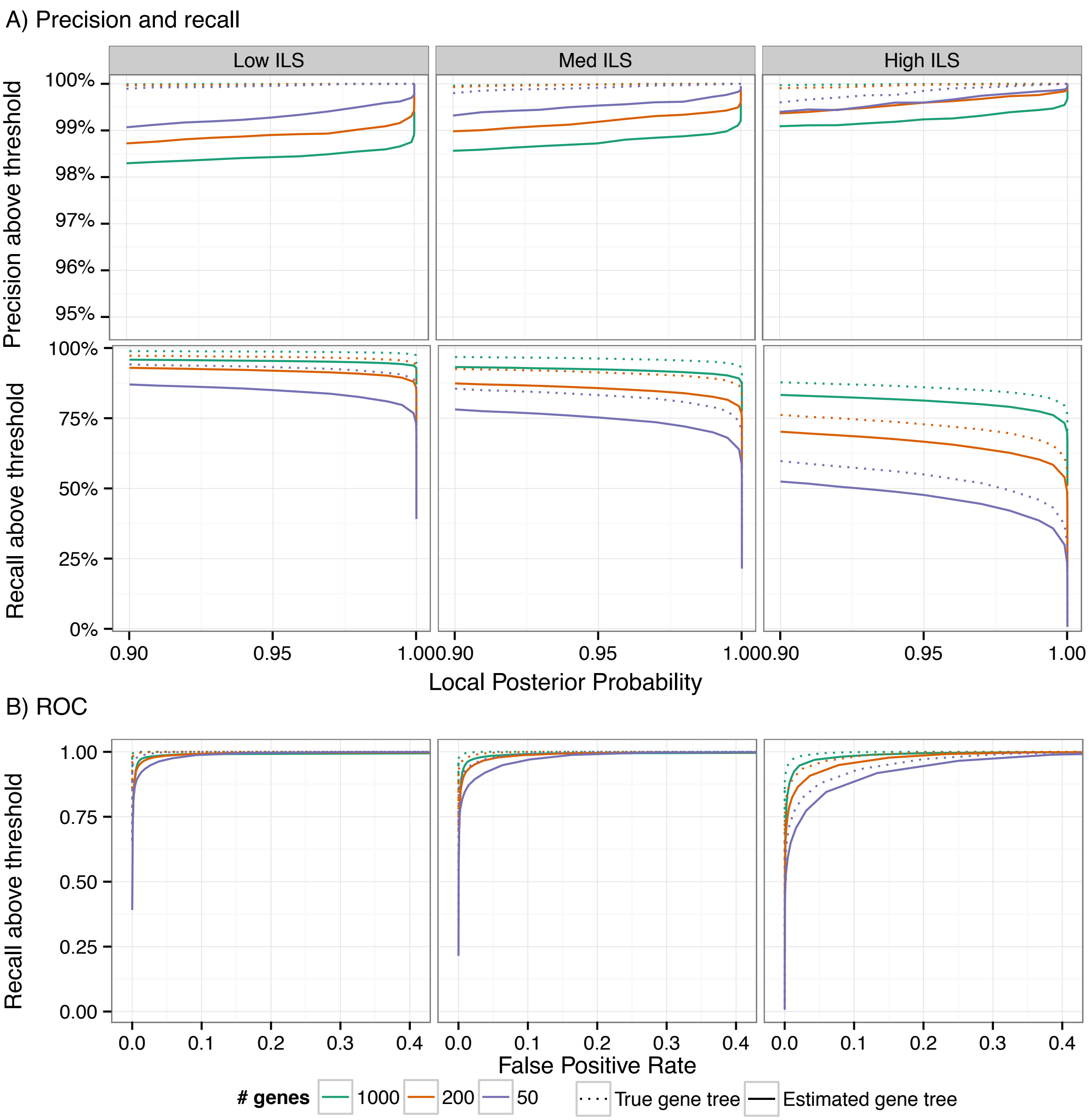

Figure S2: **Accuracy of local posterior probability on the A-200 dataset with true species trees.** A) the precision and recall of branches with local posterior probability above a threshold ranging from 0.9 to 1.0 using estimated gene trees (solid) or true gene trees (dotted). B) ROC curve (recall versus false positive rate) for thresholds ranging from 0 to 1 (figure trimmed at 0.4 fpr). Columns show different levels of ILS (each with 100 replicates). For each branch in each true species tree, all three alternatives are included in the analysis (one correct branch and two wrong branches). Thus, a total of 198*3*100=59400 branches are included for each model condition.

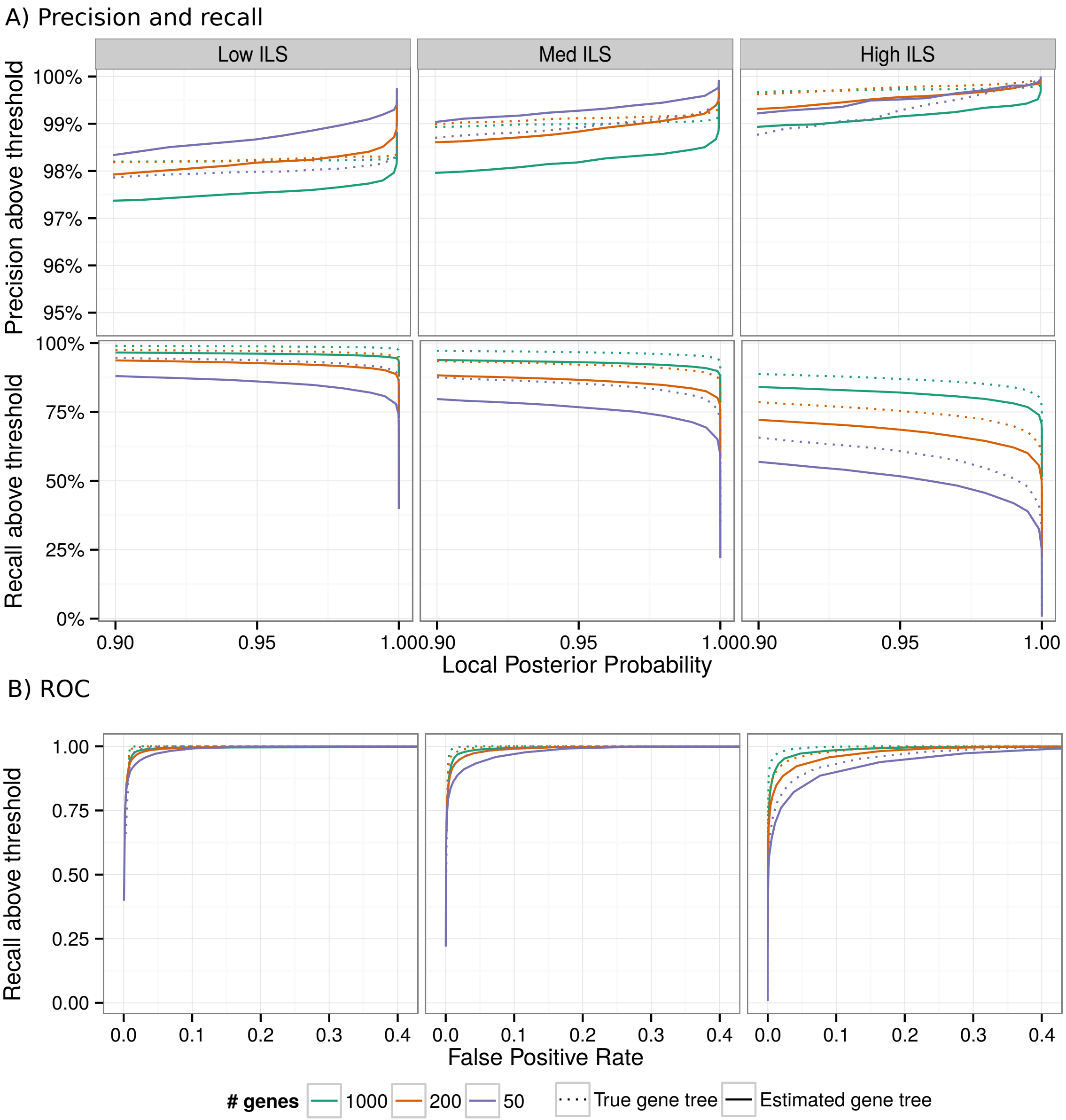

Figure S3: **Accuracy of local posterior probability on the A-200 dataset with NJST species trees.** A) the precision and recall of branches with local posterior probability above a threshold ranging from 0.9 to 1.0 using estimated gene trees (solid) or true gene trees (dotted). B) ROC curve (recall versus false positive rate) for thresholds ranging from 0 to 1 (figure trimmed at 0.4 fpr). Columns show different levels of ILS (each with 100 replicates). For each branch in each true species tree, all three alternatives are included in the analysis (one correct branch and two wrong branches). Thus, a total of 198*3*100=59400 branches are included for each model condition.

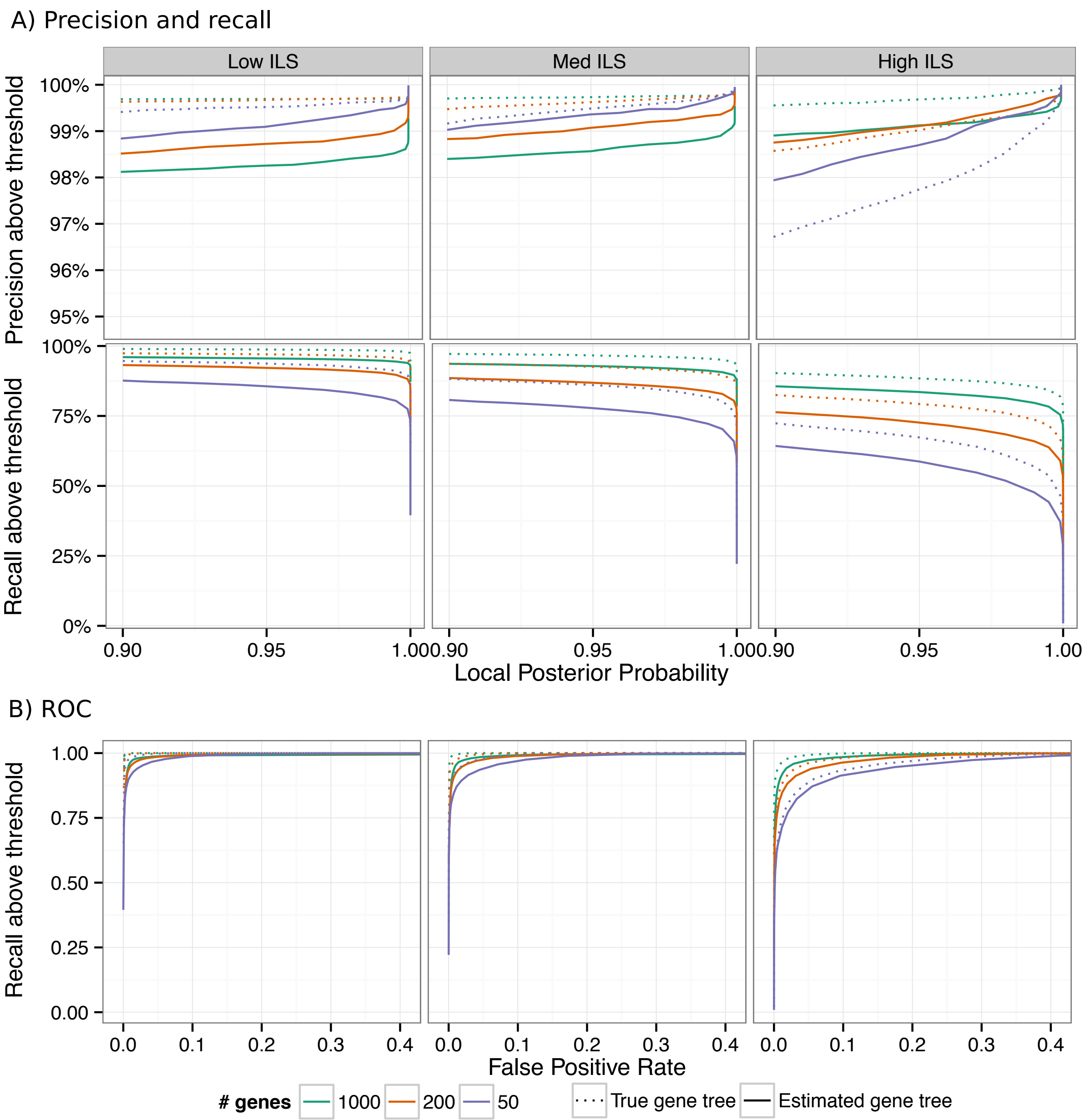

Figure S4: **Accuracy of local posterior probability on the A-200 dataset with concatenation.** A) the precision and recall of branches with local posterior probability above a threshold ranging from 0.9 to 1.0 using estimated gene trees (solid) or true gene trees (dotted). B) ROC curve (recall versus false positive rate) for thresholds ranging from 0 to 1 (figure trimmed at 0.4 fpr). Columns show different levels of ILS (each with 100 replicates). For each branch in each true species tree, all three alternatives are included in the analysis (one correct branch and two wrong branches). Thus, a total of 198*3*100=59400 branches are included for each model condition.

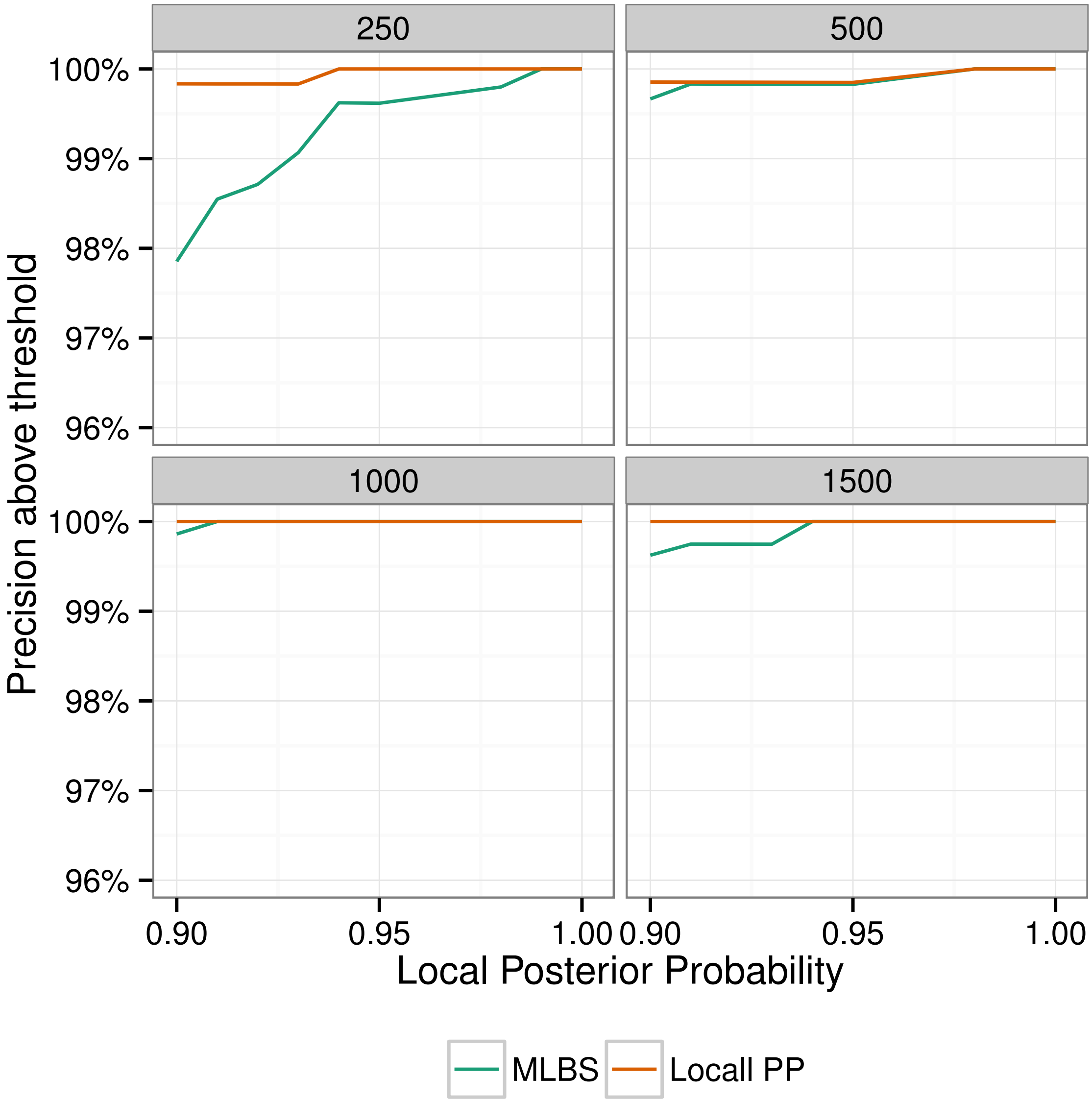

Figure S5: **Precision of local posterior probability on the Avian dataset with ASTRAL.** Precision of branches with local posterior probability above a threshold ranging from 0.9 to 1.0 based on MLBS and local posterior probability (PP) support values. Boxes show different numbers of sites per gene (controlling gene tree estimation error).

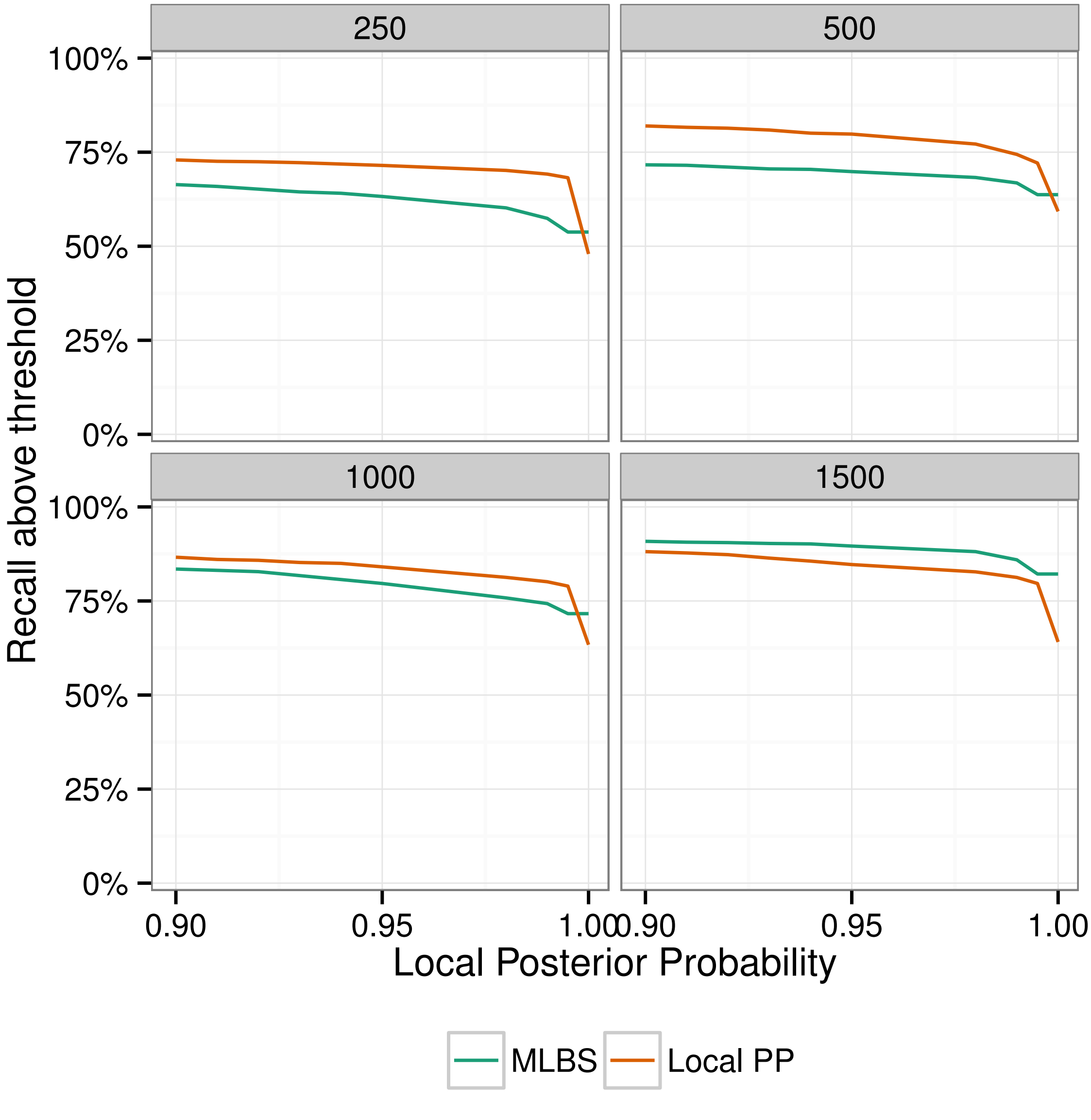

Figure S6: **Recall of local posterior probability on the Avian dataset with ASTRAL.** Recall of branches with local posterior probability above a threshold ranging from 0.9 to 1.0 based on MLBS and local posterior probability (PP) support values. Boxes show different numbers of sites per gene (controlling gene tree estimation error).

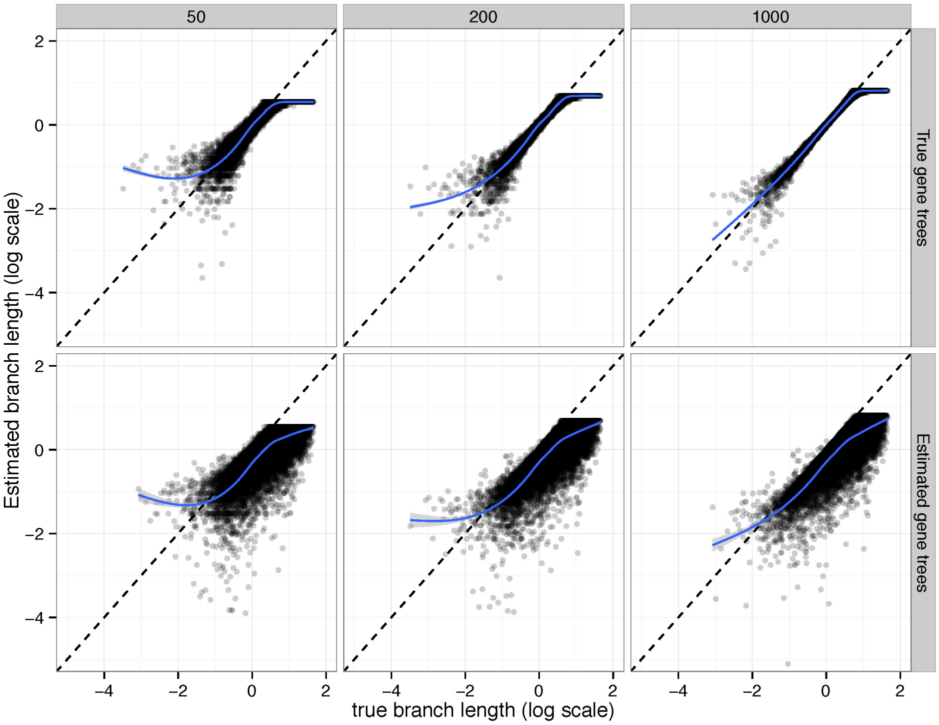

Figure S7: **Branch length accuracy on the A-200 Low ILS dataset.** Estimated branch length against true branch length is plotted in log scale (base 10). Line: fitted generalized additive model with smoothing (Wood, 2011).

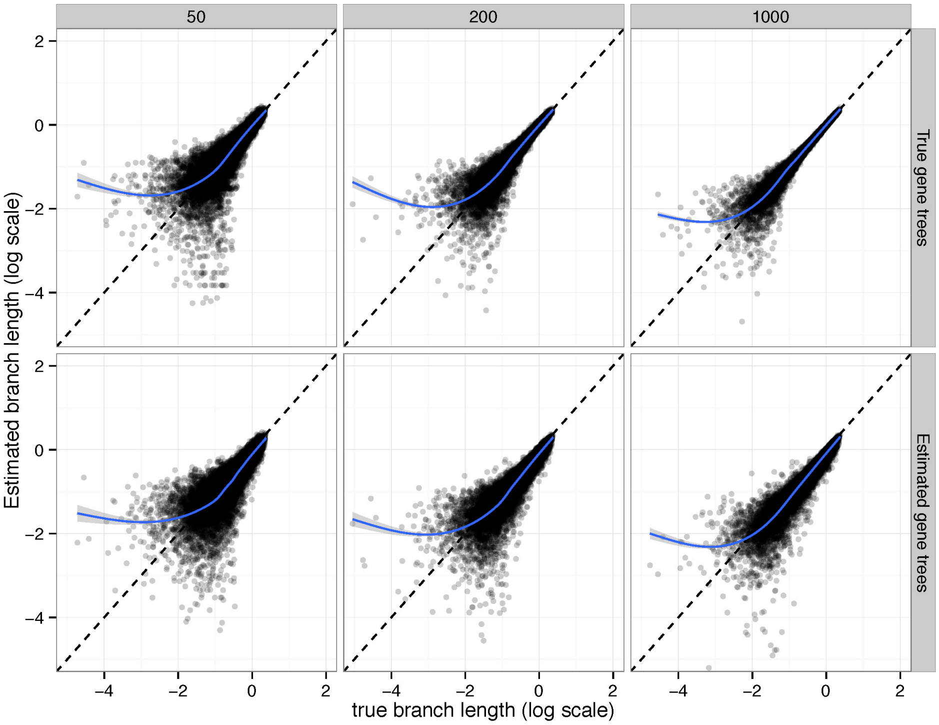

Figure S8: **Branch length accuracy on the A-200 High ILS dataset.** Estimated branch length against true branch length is plotted in log scale (base 10). Line: fitted generalized additive model with smoothing (Wood, 2011).

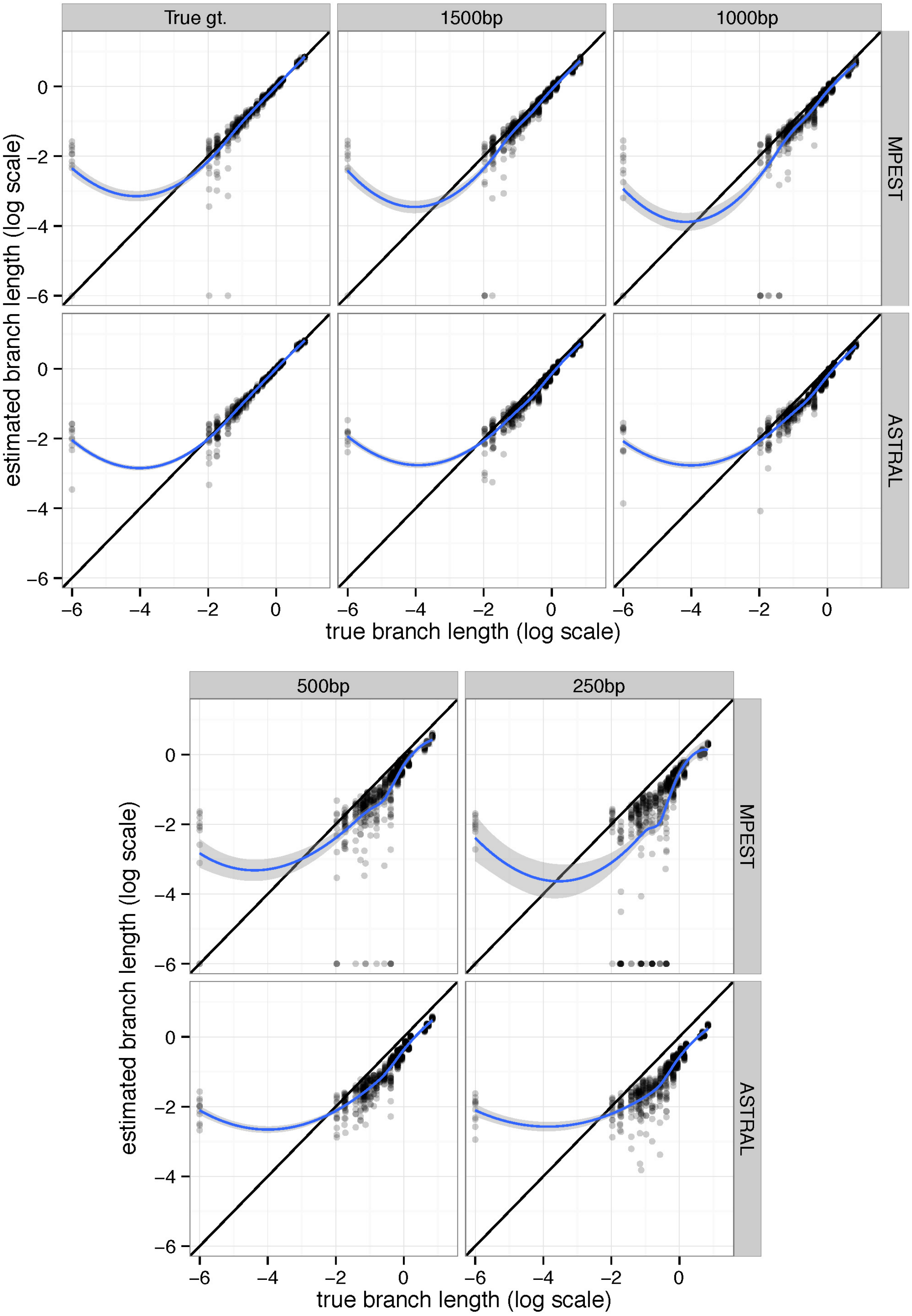

Figure S9: **Branch length accuracy on the Avian dataset.** Estimated branch length against true branch length is plotted in log scale (base 10). Line: fitted generalized additive model with smoothing (Wood, 2011).

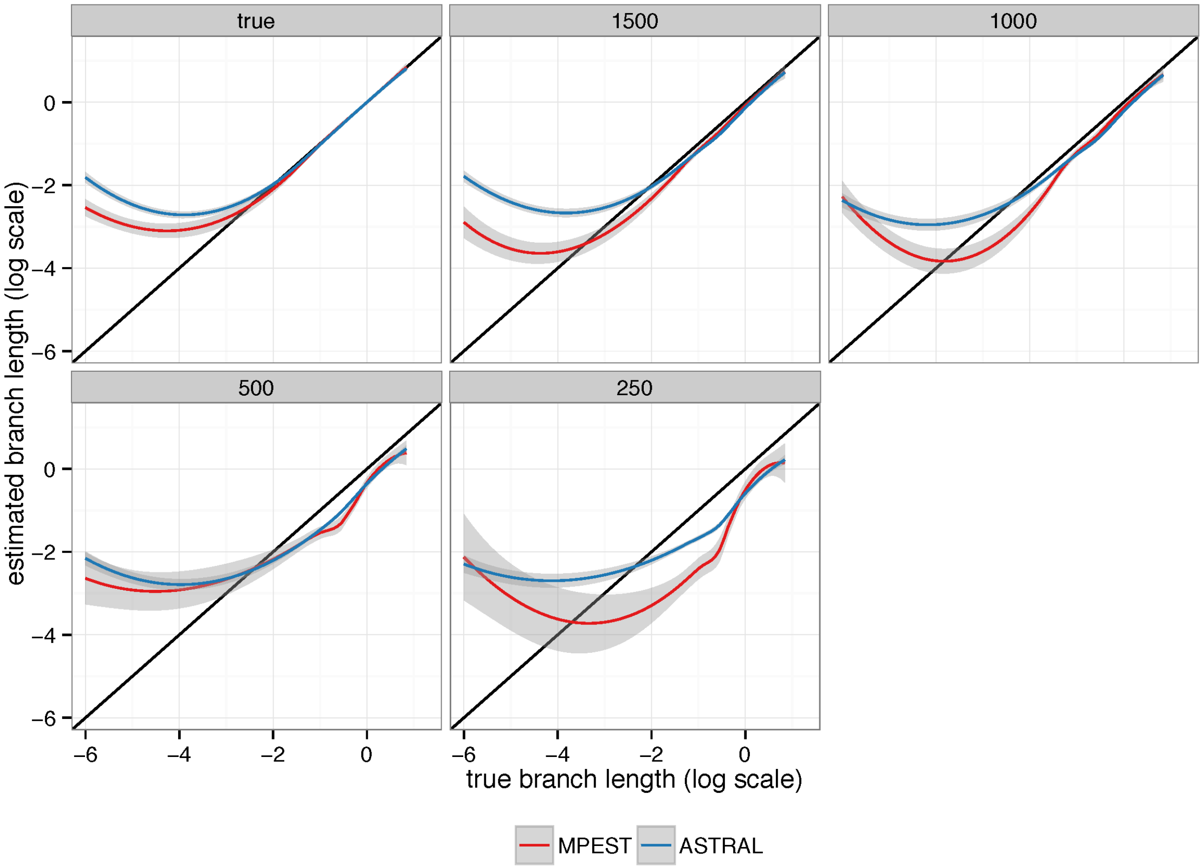

Figure S10: **Summarized branch length accuracy on the Avian dataset.** Fitted estimated branch length using MPEST, and ASTRAL methods is plotted against true branch length in log scale (base 10). Lines show a fitted generalized additive model with smoothing (Wood, 2011).

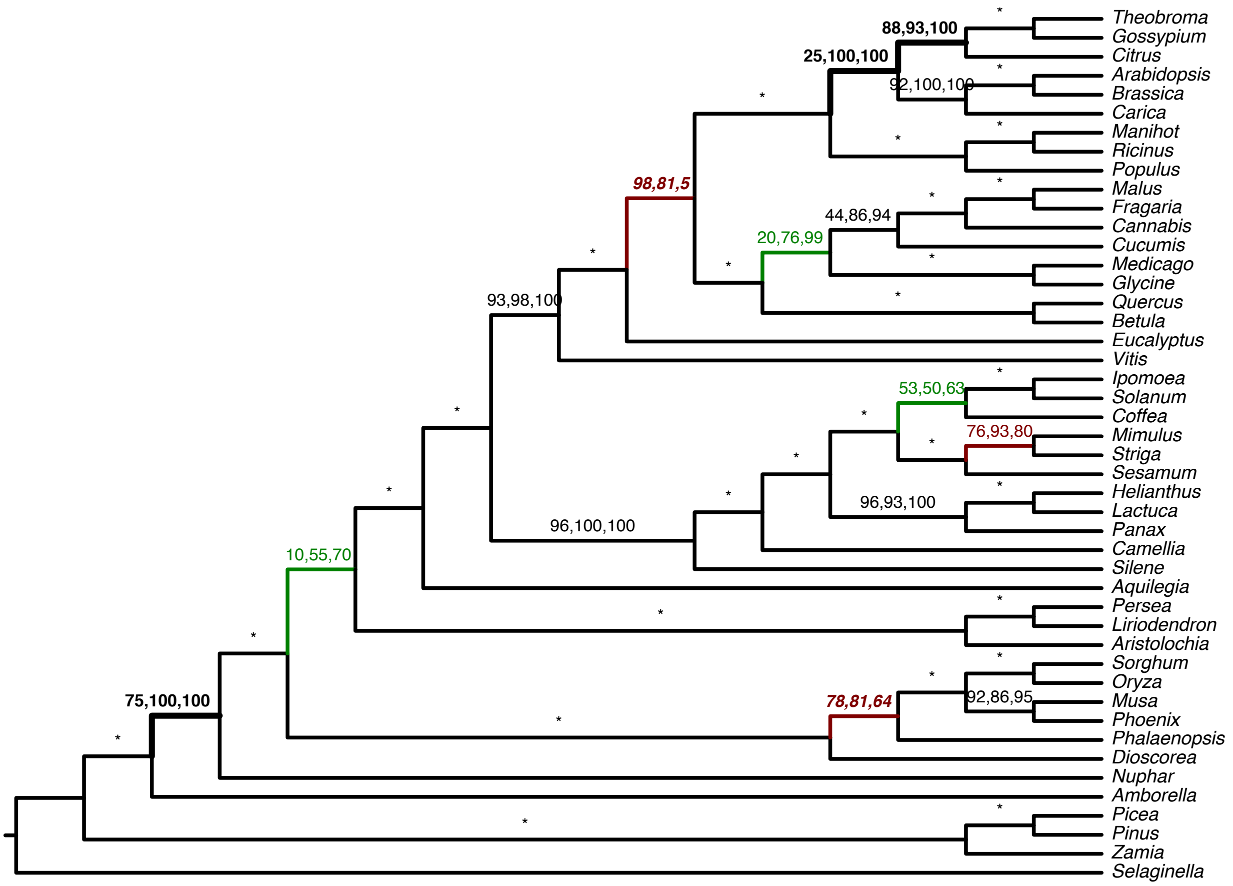

Figure S11: **Support on the angiosperm dataset** Three forms of support values are shown for the angiosperm dataset of Xi et al Xi *et al.* (2014). MLBS support (site-only), local posterior computed on fully ML resolved gene trees, and local posterior computed on ML gene trees with branches with less than 33% support collapsed. Branches marked with an asterisk (*) have 100% support with all three measures. Bold: local support on collapsed gene trees is at least 10% higher than MLBS support. Bold italic: local support on collapsed gene trees is at least 10% lower than MLBS support. Dotted/green lines (dashed/red lines): collapsing low support branches in gene trees increases (decreases) local posterior probability by at least 10%.

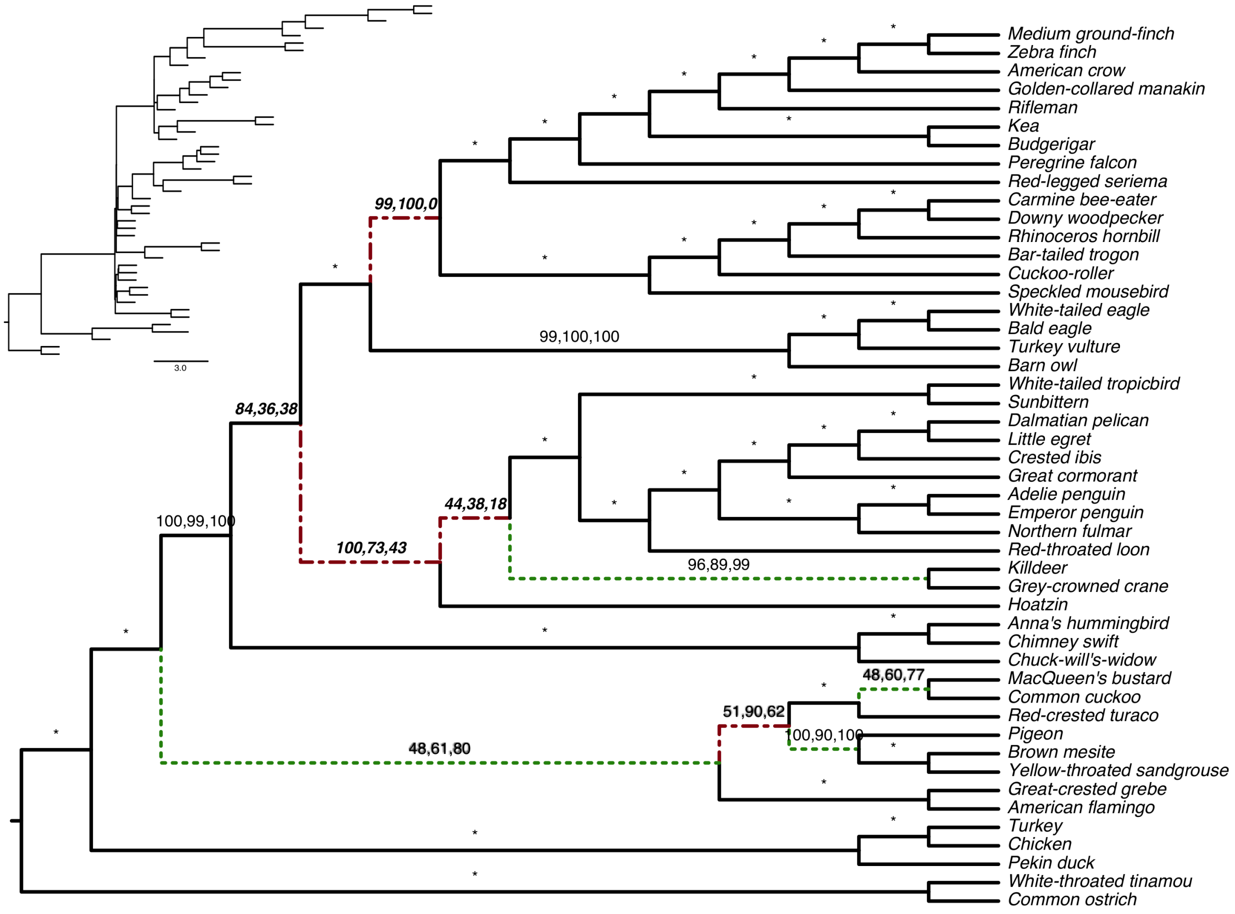

Figure S12: **Support on the avian dataset** Three forms of support values are shown for the avian dataset of Jarvis et al Jarvis *et al.* (2014). MLBS support (site-only), local posterior computed on fully ML resolved gene trees, and local posterior computed on ML gene trees with branches with less than 33% support collapsed. Branches marked with an asterisk (*) have 100% support with all three measures. Bold: local support on collapsed gene trees is at least 10% higher than MLBS support. Bold italic: local support on collapsed gene trees is at least 10% lower than MLBS support. Dotted/green lines (dashed/red lines): collapsing low support branches in gene trees increases (decreases) local posterior probability by at least 10%. Inset: branch lengths in coalescent units. The length of terminal branches are drawn arbitrarily.

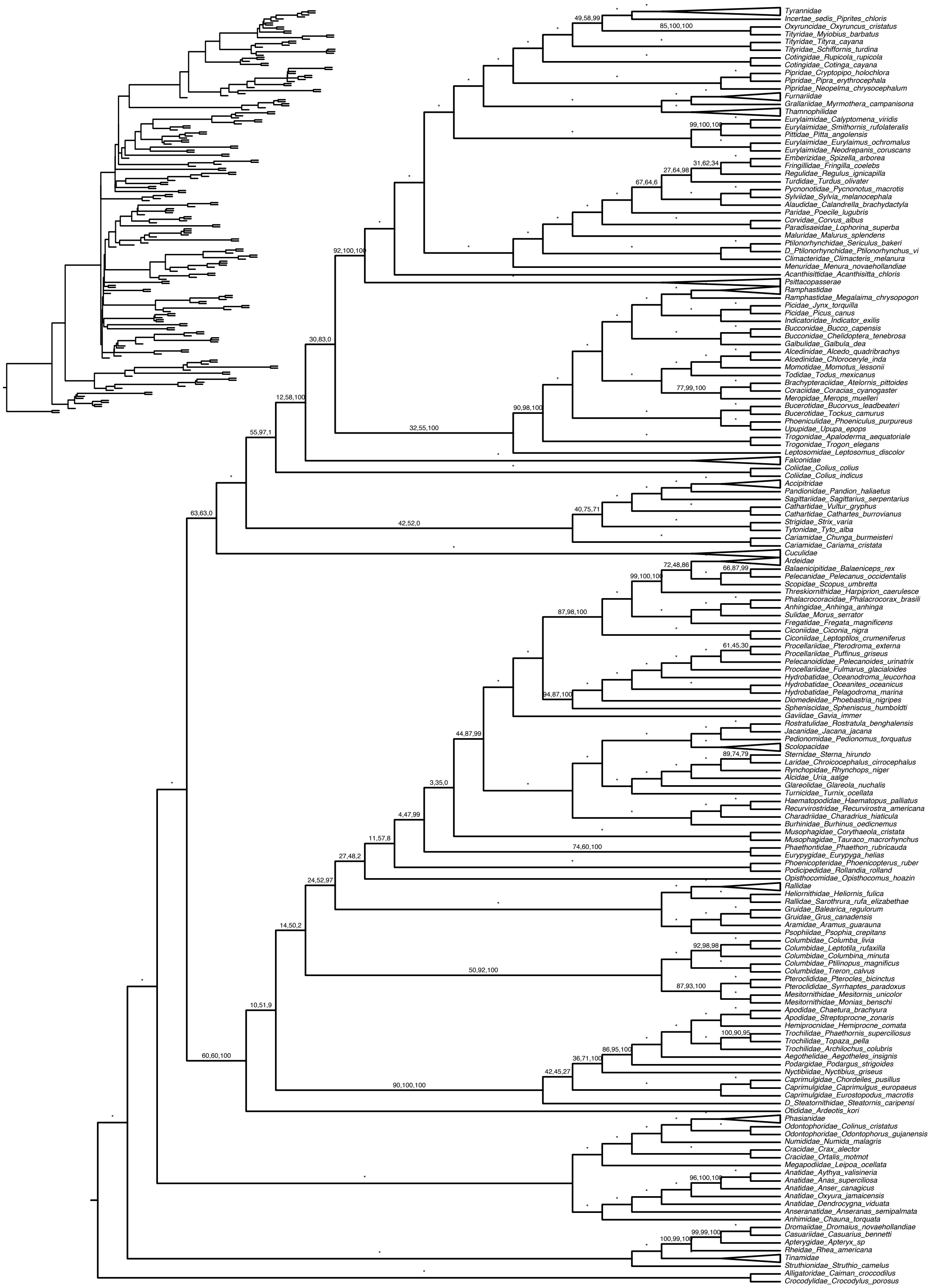

Figure S13: **Support on the avian dataset of Prum et al.** Three forms of support values are shown. Inset: branch lengths in coalescent units (terminal lengths are drawn arbitrarily). Collapsed clades had full support with all three methods for all branches.

function FREQ$(G, Q = (W, X)|(Y, Z))$
  $Q2 \leftarrow (W, Y)|(X, Z)$
  $Q3 \leftarrow (W, Z)|(X, Y)$
  $f1 \leftarrow F(G, Q)$
  $f2 \leftarrow F(G, Q2)$
  $f3 \leftarrow F(G, Q3)$
  $m \leftarrow (f1 + f2 + f3)/|G|$
  return $(f1/m, f2/m, f3/m)$

function $\mathrm{F}(G, Q = (W, X)|(Y, Z))$
  $r \leftarrow 0$
  $S \leftarrow$ empty stack
  for $g \in G$ **do**
    for $u \in postOrder(g)$ **do**
      if u is a leaf **then**
        $(w, x, y, z) \leftarrow (W[u], X[u], Y[u], Z[u])$
      else
        $(\mathbf{C}_{11}, \mathbf{C}_{12}, \mathbf{C}_{13}, \mathbf{C}_{14}) \leftarrow$ pull from S
        $(\mathbf{C}_{21}, \mathbf{C}_{22}, \mathbf{C}_{23}, \mathbf{C}_{24}) \leftarrow$ pull from S
        $(w, x, y, z) \leftarrow (\mathbf{C}_{11} + \mathbf{C}_{21}, \mathbf{C}_{12} + \mathbf{C}_{22}, \mathbf{C}_{13} + \mathbf{C}_{23}, \mathbf{C}_{14} + \mathbf{C}_{24})$
        $(\mathbf{C}_{31}, \mathbf{C}_{32}, \mathbf{C}_{33}, \mathbf{C}_{34}) \leftarrow (|W| - w, |X| - x, |Y| - y, |Z| - z)$
        $r \leftarrow r + I(\mathbf{C})$
      push (w, x, y, z) to S
  return $r/2$

function $\mathrm{I}(\mathbf{C})$
  return

$$\begin{aligned}
&\mathbf{C}_{10} \times \mathbf{C}_{21} \times \mathbf{C}_{32} \times \mathbf{C}_{33} + \mathbf{C}_{11} \times \mathbf{C}_{20} \times \mathbf{C}_{32} \times \mathbf{C}_{33} + \mathbf{C}_{12} \times \mathbf{C}_{23} \times \mathbf{C}_{30} \times \mathbf{C}_{31} + \\
&\mathbf{C}_{13} \times \mathbf{C}_{22} \times \mathbf{C}_{30} \times \mathbf{C}_{31} + \mathbf{C}_{30} \times \mathbf{C}_{21} \times \mathbf{C}_{12} \times \mathbf{C}_{13} + \mathbf{C}_{31} \times \mathbf{C}_{20} \times \mathbf{C}_{12} \times \mathbf{C}_{13} + \\
&\mathbf{C}_{32} \times \mathbf{C}_{23} \times \mathbf{C}_{10} \times \mathbf{C}_{11} + \mathbf{C}_{33} \times \mathbf{C}_{22} \times \mathbf{C}_{10} \times \mathbf{C}_{11} + \mathbf{C}_{10} \times \mathbf{C}_{31} \times \mathbf{C}_{22} \times \mathbf{C}_{23} + \\
&\mathbf{C}_{11} \times \mathbf{C}_{30} \times \mathbf{C}_{22} \times \mathbf{C}_{23} + \mathbf{C}_{12} \times \mathbf{C}_{33} \times \mathbf{C}_{20} \times \mathbf{C}_{21} + \mathbf{C}_{13} \times \mathbf{C}_{32} \times \mathbf{C}_{20} \times \mathbf{C}_{21}
\end{aligned}$$

Figure S14: **Frequency calculation algorithm.** Input is a set of gene trees G and a quadripartition $Q = (W, X)|(Y, Z)$ where each cluster (e.g., X) is a bitset indexed by the species (thus, $X[u]$ is 1 if leaf u is in X and otherwise is 0). Output is the quartet frequency for each of the three topologies around that branch.

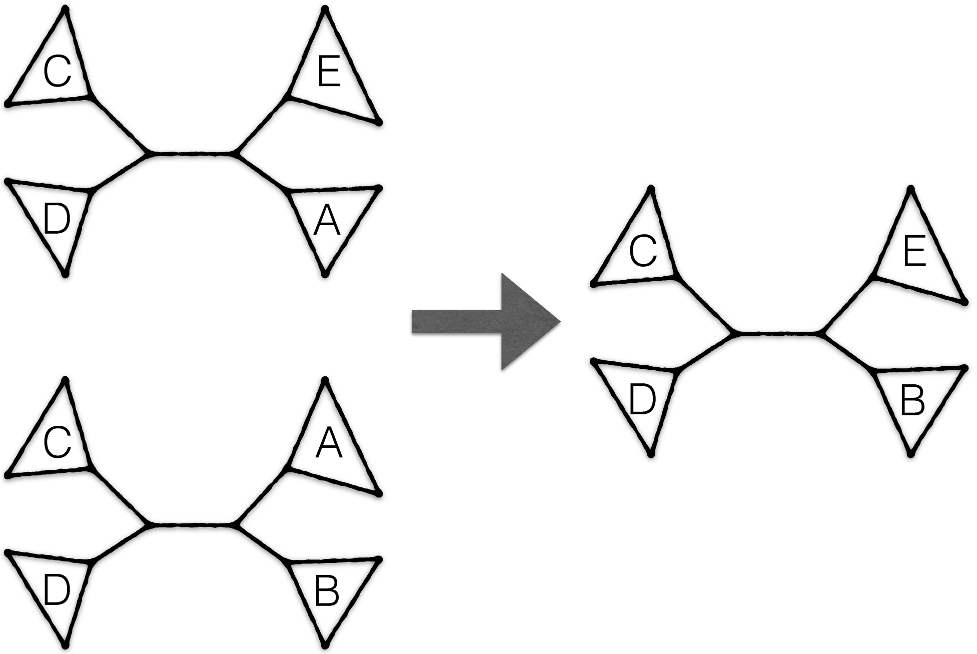

Figure S15: **Topologies of different quartets around a branch might heavily depend on each other** In this example, species A and B and E are closer to each other than each of them to C or D. Lets assume we observe topology of set of taxa A, B, C, D is $T_1 = A, B|C, D$ in a gene tree G, and the topology of set of taxa A, E, C, D is $T_2 = A, E|C, D$ in G. In this case the topology of set of taxa $T_3 = B, E|C, D$ is completely determined based on topologies T_1, and T_2. So it does not have extra information about the internal branch between them. To see this, imagine putting B on each of the five branches of $A, E|C, D$. Two branches (pending to C and B are not possible, because they contradict the other quartet topology. The other three placements all results in the $B, E|C, D$ topology.

2 Supplementary methods

3 Proofs

3.1 Proof of Lemma 1

Lemma 1 *Let* $(\theta_1, \theta_2, \theta_3)$ *denote parameters of the true multinomial distribution generating* $\bar{Z}$*. Note* $\sum_1^3 \theta_i = 1$ *and the two lower* θ_i*s are identical, and recall* z_1 *corresponds to the topology of* Q*.*

$$P(\theta_1 > \frac{1}{3}|\bar{Z} = \bar{z}) = \frac{\int_{\frac{1}{3}}^{1} P(\bar{Z} = \bar{z}|\theta_1 = t) f_{\theta_1}(t)\mathrm{d}t}{P(\bar{Z} = \bar{z})} \tag{S1}$$

with likelihood term:

$$P(\bar{Z} = \bar{z}|\theta_1 = t) = \Gamma t^{z_1} (\frac{1-t}{2})^{n-z_1} \tag{S2}$$

where $\Gamma = \frac{\Gamma(n+1)}{\prod_1^3 \Gamma(z_j+1)}$*, and marginal probability:*

$$\begin{aligned} P(\bar{Z} = \bar{z}) &= \sum_{j=1}^{3} \int_{\frac{1}{3}}^{1} P(\bar{Z} = \bar{z}|\theta_j = t) f_{\theta_j}(t)\mathrm{d}t \\ &= \Gamma \sum_{j=1}^{3} \int_{\frac{1}{3}}^{1} t^{z_j} (\frac{1-t}{2})^{n-z_j} f_{\theta_j}(t)\mathrm{d}t. \end{aligned} \tag{S3}$$

Proof To prove Equation (S1), one could use Bayes' rule directly,

$$\begin{aligned} P(\theta_1 > \frac{1}{3}|\bar{Z} = \bar{z}) = \frac{P(\theta_1 > \frac{1}{3}, \bar{Z} = \bar{z})}{P(\bar{Z} = \bar{z})} &= \frac{\int_{\frac{1}{3}}^{1} P(\bar{Z} = \bar{z}, \theta_1 = t)\mathrm{d}t}{P(\bar{Z} = \bar{z})} \\ &= \frac{\int_{\frac{1}{3}}^{1} P(\bar{Z} = \bar{z}|\theta_1 = t) f_{\theta_1}(t)\mathrm{d}t}{P(\bar{Z} = \bar{z})} \end{aligned}$$

Equation (S2) comes from $\bar{Z}$ being a multinomial distributed random variable given $\theta_1 = t$ is fixed and $\theta_1 > \frac{1}{3}$ (so $\theta_2 = \theta_3 = \frac{1-t}{2}$) directly. To prove Equation (S3),

$$\begin{aligned} P(\bar{Z} = \bar{z}) =& P(\bar{Z} = \bar{z}|\theta_1 > \frac{1}{3}) + P(\bar{Z} = \bar{z}|\theta_1 \leq \frac{1}{3}) = P(\bar{Z} = \bar{z}|\theta_1 > \frac{1}{3}) + P(\bar{Z} = \bar{z}|\theta_2 > \frac{1}{3} \vee \theta_3 > \frac{1}{3}) \\ =& P(\bar{Z} = \bar{z}|\theta_1 > \frac{1}{3}) + P(\bar{Z} = \bar{z}|\theta_2 > \frac{1}{3}) + P(\bar{Z} = \bar{z}|\theta_3 > \frac{1}{3}) \end{aligned}$$

3.2 Proof of Theorem 1

Theorem *Given 1) a set of* n *gene trees generated by the MSC on a model species tree generated by the Yule process with rate* λ *and 2) an internal branch represented by a quadripartition* Q *where the four clusters around* Q *are each present in the species tree, let* $\bar{z} = (z_1, z_2, z_3)$ *be the average quartet frequencies around* Q *(where* z_1 *corresponds to the topology of* Q*); the local posterior probability that the species tree has the topology given by* Q *is:*

$$P(Q|\bar{Z} = \bar{z}) = \frac{h(z_1)}{h(z_1) + 2^{z_2 - z_1} h(z_2) + 2^{z_3 - z_1} h(z_3)} \tag{S4}$$

where

$$h(x) = \mathbf{B}(x+1, n-x+2\lambda)(1 - I_{\frac{1}{3}}(x+1, n-x+2\lambda)).$$

Here, $\mathbf{B}(\alpha, \beta)$ *is the beta function, and* I_x *is the regularized incomplete beta function.*

Proof $\bar{Z}$ follows a multinomial distribution with parameters $(\theta_1, \theta_2, \theta_3)$. Lack of anomaly zones for unrooted quartets, shown by Allman *et al.* (2011) means that Q is in the species tree iff $\theta_1 > \frac{1}{3}$. Thus, by Lemma 1 we can use (S1), (S2), and (S3) to compute the local posterior probability of Q. By the assumption that (coalescent unit) branch lengths in the species tree are generated by the Yule process, calculation of (S4) follows from manipulating (S1), (S2), and (S3), as detailed in the following Using Lemma 1,

$$P(\theta_1 > \frac{1}{3}|\bar{Z} = \bar{z}) = \frac{\int_{\frac{1}{3}}^{1} P(\bar{Z} = \bar{z}|\theta_1 = t) f_{\theta_1}(t) \mathrm{d}t}{P(\bar{Z} = \bar{z})}$$

with likelihood term:

$$P(\bar{Z} = \bar{z}|\theta_1 = t) = \Gamma t^{z_1} (\frac{1-t}{2})^{n-z_1}$$

where $\Gamma = \frac{\Gamma(n+1)}{\prod_1^3 \Gamma(z_j+1)}$, and marginal probability:

$$\begin{aligned} P(\bar{Z} = \bar{z}) &= \sum_{j=1}^{3} \int_{\frac{1}{3}}^{1} P(\bar{Z} = \bar{z}|\theta_j = t) f_{\theta_j}(t) \mathrm{d}t \\ &= \Gamma \sum_{j=1}^{3} \int_{\frac{1}{3}}^{1} t^{z_j} (\frac{1-t}{2})^{n-z_j} f_{\theta_j}(t) \mathrm{d}t. \end{aligned}$$

In these equations $\bar{Z}$ is a multinomial random variable with parameters $(\theta_1, \theta_2, \theta_3)$, $\sum_1^3 \theta_i = 1$ and the two lower θ_is are identical, and recall z_1 corresponds to the topology of Q. Using (S3), and (S1),

$$P(Q|\bar{Z} = \bar{z}) = \frac{\int_{\frac{1}{3}}^{1} P(\bar{Z} = \bar{z}|\theta_1 = t) f_{\theta_1}(t) \mathrm{d}t}{\Gamma \sum_{j=1}^{3} \int_{\frac{1}{3}}^{1} g(z_j; n, t) f_{\theta_j}(t) \mathrm{d}t} \tag{S5}$$

Based on Lemma 2, and Equation S2 $f_{\theta_j}(t) = \lambda(\frac{3}{2}(1-t))^{2\lambda-1}$, and $P(\bar{Z} = \bar{z}|\theta_j = t) = \Gamma t^{z_j} (\frac{1-t}{2})^{n-z_j}$. So Equation S5 simplifies to:

$$\begin{aligned} P(Q|\bar{Z} = \bar{z}) =& \frac{\int_{\frac{1}{3}}^{1} (\Gamma t^{z_1} (\frac{1-t}{2})^{n-z_1}) \lambda(\frac{3}{2}(1-t))^{2\lambda-1} \mathrm{d}t}{\Gamma \sum_{j=1}^{3} \int_{\frac{1}{3}}^{1} (t^{z_j} (\frac{1-t}{2})^{n-z_j}) \lambda(\frac{3}{2}(1-t))^{2\lambda-1} \mathrm{d}t} \\ =& \frac{2^{z_1-n} \int_{\frac{1}{3}}^{1} t^{z_1} (1-t)^{n-z_1+2\lambda-1} \mathrm{d}t}{\sum_{j=1}^{3} 2^{z_j-n} \int_{\frac{1}{3}}^{1} t^{z_j} (1-t)^{n-z_j+2\lambda-1} \mathrm{d}t} \\ =& \frac{2^{z_1-n} (\int_0^1 t^{z_1} (1-t)^{n-z_1+2\lambda-1} \mathrm{d}t - \int_0^{\frac{1}{3}} t^{z_1} (1-t)^{n-z_1+2\lambda-1} \mathrm{d}t)}{\sum_{j=1}^{3} 2^{z_j-n} (\int_0^1 t^{z_j} (1-t)^{n-z_j+2\lambda-1} \mathrm{d}t - \int_0^{\frac{1}{3}} t^{z_j} (1-t)^{n-z_j+2\lambda-1} \mathrm{d}t)} \end{aligned} \tag{S6}$$

With $\mathbf{B}(\alpha, \beta)$ as beta function, and I_x as the regularized incomplete beta function,

$$\mathbf{B}(x+1, n-x+2\lambda) = \int_0^1 t^{z_j} (1-t)^{n-z_j+2\lambda-1} \mathrm{d}t$$

and,

$$\mathbf{B}(x+1, n-x+2\lambda) I_{\frac{1}{3}}(x+1, n-x+2\lambda) = \int_0^{\frac{1}{3}} t^{z_j} (1-t)^{n-z_j+2\lambda-1} \mathrm{d}t$$

then,

$$P(Q|\bar{Z} = \bar{z}) = \frac{h(z_1)}{h(z_1) + 2^{z_2 - z_1} h(z_2) + 2^{z_3 - z_1} h(z_3)} \tag{S7}$$

where

$$h(x) = \mathbf{B}(x+1, n-x+2\lambda)(1 - I_{\frac{1}{3}}(x+1, n-x+2\lambda)).$$

3.3 Proof of Theorem 2

Theorem *Under conditions of Theorem 1, and assuming the branch represented by Q is in the species tree, the ML estimate for its length is* $-\ln \frac{3}{2}(1-\frac{z_1}{n})$ *and the MAP estimate is* $-\ln \frac{3}{2}(1-\frac{z_1}{n+2\lambda})$ *when* $3z_1 \geq n$*; otherwise, both ML and MAP are zero.*

Proof Assume D, branch length, is a random variable whose range is in $[0\ \infty)$. The ML estimate for the branch length is coming from:

$$d^*_{ML} = \underset{x \geq 0}{\operatorname{argmax}}\, P_{Z_1|D}(z_1|x; n) \tag{S8}$$

where $P_{Z_1|D}(z_1|x;n)$ is the likelihood of D. Assuming that Q is the true topology, the likelihood of D is proportional to:

$$P_{Z_1|D}(z_1|x;n) \propto (1 - \frac{2}{3}e^{-x})^{z_1} (\frac{1}{3}e^{-x})^{n-z_1} \tag{S9}$$

computing the log likelihood and removing the constants yields to

$$L(x; z, n) = z_1 \ln(1 - \frac{2}{3}e^{-x}) - (n - z_1)x. \tag{S10}$$

Note that

$$\frac{\mathrm{d}^2 L(x;z,n)}{\mathrm{d}x^2} = \frac{-z_1 \frac{2}{3} e^{-x}}{(1 - \frac{2}{3}e^{-x})^2} < 0, \tag{S11}$$

so (S10) is a concave function. To find the maximum of (S10), we take its derivative and set it to zero. Let's name the solution as $\hat{d}$

$$\frac{\mathrm{d}L(x;z,n)}{\mathrm{d}x} = \frac{z_1 \frac{2}{3} e^{-x}}{1 - \frac{2}{3}e^{-x}} - (n - z_1) = 0. \tag{S12}$$

Due to concavity of (S10), and condisering (S8) $d^*_{ML} = \max(0, \hat{d})$. The solution of (S12) is $\hat{d} = -\ln(\frac{3}{2}(1 - \frac{z_1}{n}))$. Note that $\hat{d} \geq 0$ if $\frac{3}{2}(1 - \frac{z_1}{n}) \leq 1$ or $\frac{z_1}{n} \geq \frac{1}{3}$. So $d^*_{ML} = -\ln(\frac{3}{2}(1 - \frac{z_1}{n}))$ if $\frac{z_1}{n} \geq \frac{1}{3}$, otherwise $d^*_{ML} = 0$.

Given n gene trees, to find the MAP estimate we need to solve

$$\begin{aligned} D^*_{MAP} &= \underset{x \geq 0}{\operatorname{argmax}}\, f_{D|Z_1}(x|z_1; n) \\ &= \underset{x \geq 0}{\operatorname{argmax}}\, P_{Z_1|D}(z_1|x) f_D(x) \end{aligned} \tag{S13}$$

where $f_D(x)$ is a priori distribution for branch length.

The waiting time between the two consecutive speciation events is the length of the interior edge between them. Assuming that our species tree is a Yule tree, each species has a speciation rate λ, and this waiting time is exponentially distributed with mean $\frac{1}{2\lambda}$ (Stadler and Steel, 2012).

So for the MAP estimate, we assume branch length D is exponentially distributed with rate 2λ. In this case the posteriori could be written as:

$$f_{D|Z_1}(x|z_1;n) \propto (1-\frac{2}{3}e^{-x})^{z_1}(\frac{1}{3}e^{-x})^{n-z_1}e^{-2\lambda x}. \tag{S14}$$

Taking log of (S14) and removing constants the function $L'(x)$ is defined as

$$L'(x) = z_1 \ln(1-\frac{2}{3}e^{-x}) - (n - z_1 + 2\lambda). \tag{S15}$$

Note that

$$\frac{\mathrm{d}^2 L'(x;z,n)}{\mathrm{d}x^2} = \frac{-z_1\frac{2}{3}e^{-x}}{(1-\frac{2}{3}e^{-x})^2} < 0, \tag{S16}$$

so (S15) is a concave function. To find the maximum of (S15), we take its derivative and set it to zero. Lets name the solution as $\hat{d}'$

$$\frac{\mathrm{d}L'(x)}{dx} = \frac{z_1\frac{2}{3}e^{-x}}{1-\frac{2}{3}e^{-x}} - (n - z_1 + 2\lambda) = 0. \tag{S17}$$

Due to concavity of (S15) and following (S13) the MAP estimate is $d^*_{MAP} = \max(0, \hat{d}')$. Solving (S17) $\hat{d}' = -\ln(\frac{3}{2}(1-\frac{z_1}{n+2\lambda}))$. Note that $\hat{d}' \geq 0$ if $\frac{3}{2}(1-\frac{z_1}{n+2\lambda}) \leq 1$ or $\frac{z_1}{n+2\lambda} \geq \frac{1}{3}$. So, $d^*_{MAP} = -\ln(\frac{3}{2}(1-\frac{z_1}{n+2\lambda}))$ if $\frac{z_1}{n+2\lambda} \geq \frac{1}{3}$, otherwise the MAP estimate is $d^*_{MAP} = 0$.

3.4 Proof of Lemma 2

Lemma *If the species tree is generated using the Yule process with rate λ, branch lengths are exponentially distributed, and for $t \geq \frac{1}{3}$:*

$$f_{\theta_j}(t) = \lambda(3\frac{1-t}{2})^{2\lambda-1} \tag{S18}$$

Proof Under the Yule process with rate λ, the branch lengths D are exponentially distributed with rate 2λ, and the PDF of D is $f_D(x) = 2\lambda e^{-2\lambda x}$ (Stadler and Steel, 2012). Note that because of absence of extra reliable information about the species tree topology, we use an uninformative prior, which means all topologies are equally likely to be the dominant one ($Pr(\theta_1 > \frac{1}{3}) = Pr(\theta_2 > \frac{1}{3}) = Pr(\theta_3 > \frac{1}{3}) = \frac{1}{3}$). Knowing that $\theta_j = 1 - \frac{2}{3}e^{-x}$ is in $[\frac{1}{3}, 1)$ as x goes from 0 to ∞ ($j = 1, 2, 3$), and by using the transformation rule of random variables:

$$f_{\theta_j}(t) = \frac{1}{3}\frac{1}{|\frac{d\theta_j}{dx}|}f_D(x)\Big|_{x=-\ln((1-t)\frac{3}{2})} = \lambda e^{(-2\lambda+1)x}\Big|_{x=-\ln((1-t)\frac{3}{2})} = \lambda((1-t)\frac{3}{2})^{2\lambda-1}. \tag{S19}$$

4 Commands and version numbers

4.1 ASTRAL

We used ASTRAL version 4.9.1 available at `https://github.com/smirarab/ASTRAL/tree/posteval` for scoring. We also used ASTRAL version 4.9.8 for computing the branch lengths of the trees. To have posterior probabilities of branches of main species tree and 2 other alternatives we used:

```
java -Xmx2000M -jar astral.4.9.1.jar -i [GENE TREES] -q [SPECIES TREE] -t 4
```

To compute the branch lengths of main species tree we used the MAP estimate with the command:

```
java -Xmx2000M -jar astral.4.9.8.jar -i [GENE TREES] -q [SPECIES TREE] -t 2
```

Users can find the most updated codes available at `https://github.com/smirarab/ASTRAL/`. To score and compute the branch lengths, and local posterior probabilities of inferred species tree one can use:

```
java -Xmx2000M -jar astral.4.10.0 -i [GENE TREES] -q [SPECIES TREE] -t 3
```

4.2 MP-EST

MP-EST version 1.5 was used for estimating branch lengths on a fixed topology. We used a custom shell script to run MP-EST 2 times with different random seed numbers and take the tree with the highest likelihood. The shell script is available at `https://github.com/smirarab/global/tree/master/src/shell`.

References

Allman, E. S., Degnan, J. H., and Rhodes, J. A. 2011. Identifying the rooted species tree from the distribution of unrooted gene trees under the coalescent. *J. Math. Biol.*, 62: 833–862.

Jarvis, E. D., Mirarab, S., Aberer, A. J., Li, B., Houde, P., Li, C., Ho, S. Y. W., Faircloth, B. C., Nabholz, B., Howard, J. T., Suh, A., Weber, C. C., da Fonseca, R. R., Li, J., Zhang, F., Li, H., Zhou, L., Narula, N., Liu, L., Ganapathy, G., Boussau, B., Bayzid, M. S., Zavidovych, V., Subramanian, S., Gabaldón, T., Capella-Gutiérrez, S., Huerta-Cepas, J., Rekepalli, B., Munch, K., Schierup, M. H., Lindow, B., Warren, W. C., Ray, D., Green, R. E., Bruford, M. W., Zhan, X., Dixon, A., Li, S., Li, N., Huang, Y., Derryberry, E. P., Bertelsen, M. F., Sheldon, F. H., Brumfield, R. T., Mello, C. V., Lovell, P. V., Wirthlin, M., Schneider, M. P. C., Prosdocimi, F., Samaniego, J. A., Velazquez, A. M. V., Alfaro-Núñez, A., Campos, P. F., Petersen, B., Sicheritz-Ponten, T., Pas, A., Bailey, T., Scofield, P., Bunce, M., Lambert, D. M., Zhou, Q., Perelman, P., Driskell, A. C., Shapiro, B., Xiong, Z., Zeng, Y., Liu, S., Li, Z., Liu, B., Wu, K., Xiao, J., Yinqi, X., Zheng, Q., Zhang, Y., Yang, H., Wang, J., Smeds, L., Rheindt, F. E., Braun, M. J., Fjeldsa, J., Orlando, L., Barker, F. K., Jønsson, K. A., Johnson, W., Koepfli, K.-P., O'Brien, S., Haussler, D., Ryder, O. A., Rahbek, C., Willerslev, E., Graves, G. R., Glenn, T. C., McCormack, J. E., Burt, D. W., Ellegren, H., Alström, P., Edwards, S. V., Stamatakis, A., Mindell, D. P., Cracraft, J., Braun, E. L., Warnow, T., Jun, W., Gilbert, M. T. P., and Zhang, G. 2014. Whole-genome analyses resolve early branches in the tree of life of modern birds. *Science*, 346(6215): 1320–1331.

Mirarab, S. and Warnow, T. 2015. ASTRAL-II: coalescent-based species tree estimation with many hundreds of taxa and thousands of genes. *Bioinformatics*, 31(12): i44–i52.

Robinson, D. and Foulds, L. 1981. Comparison of phylogenetic trees. *Mathematical Biosciences*, 53(1-2): 131–147.

Stadler, T. and Steel, M. 2012. Distribution of branch lengths and phylogenetic diversity under homogeneous speciation models. *Journal of Theoretical Biology*, 297: 33–40.

Wood, S. N. 2011. Fast stable restricted maximum likelihood and marginal likelihood estimation of semiparametric generalized linear models. *Journal of the Royal Statistical Society: Series B (Statistical Methodology)*, 73(1): 3–36.

Xi, Z., Liu, L., Rest, J. S., and Davis, C. C. 2014. Coalescent versus Concatenation Methods and the Placement of Amborella as Sister to Water Lilies. *Systematic Biology*, 63(6): 919–932.
